# Changes in statin therapy eligibility under the 2026 versus 2018 dyslipidemia guidelines

**DOI:** 10.1016/j.ajpc.2026.101719

**Published:** 2026-06-26

**Authors:** Jared A. Spitz, Faith E. Metlock, Bede Nriagu, Carl Ade, Britton Scheuermann, Lily Dastmalchi, Garima Sharma, Samuel Kim

**Affiliations:** a Inova Schar Heart and Vascular, Inova Health System, Falls Church, VA, USA; b College of Health and Human Sciences, Kansas State University, Manhattan, USA; c Department of Cardiology, NewYork-Presbyterian Hospital/Weill Cornell Medicine, New York, NY, USA

**Keywords:** Atherosclerotic cardiovascular disease, Dyslipidemia, Statin eligibility, PREVENT-ASCVD, Primary prevention

## Abstract

**Background:**

The 2026 ACC/AHA Dyslipidemia Guideline replaced the 2018 guideline and adopted PREVENT-ASCVD as the preferred primary prevention risk framework. Although prior analyses applying PREVENT-ASCVD alone suggested that statin eligibility may decrease because of lower absolute risk estimates compared with the Pooled Cohort Equations, the population-level impact of the full 2026 guideline framework remains unclear.

**Methods:**

We analyzed adults from the 2017–2020 National Health and Nutrition Examination Survey to assess statin eligibility under the 2018 and 2026 ACC/AHA guideline frameworks. Survey weights generated nationally representative estimates. Participants were classified as eligible under both guidelines, eligible only under the 2018 guideline, eligible only under the 2026 guideline, or ineligible under both. We estimated overall and subgroup-specific changes in eligibility and used logistic regression to identify factors associated with new eligibility under the 2026 guideline.

**Results:**

Among a weighted population of 77.8 million U.S. adults aged 30–79 years (N = 2747), statin eligibility increased from 39.8% under the 2018 guideline to 54.3% under the 2026 guideline, representing 11.2 million additional eligible adults and a 36.2% relative increase. Most adults were classified similarly under both guidelines, while 16.5% were newly eligible and 2.1% lost eligibility. The largest relative increase occurred among adults younger than 50 years. Greater age, sex, greater body mass index, higher total cholesterol, lower eGFR, current smoking, and hypertension were associated with new eligibility.

**Conclusions:**

The 2026 ACC/AHA Dyslipidemia Guideline substantially expanded statin eligibility, reflecting the full updated eligibility framework rather than PREVENT-ASCVD alone. Expanded eligibility will require implementation strategies to improve statin access, uptake, and long-term ASCVD prevention.

## 1 Introduction

Atherosclerotic cardiovascular disease (ASCVD) is the leading cause of death in the United States, and dyslipidemia is among its most consequential modifiable risk factors [1,2]. Statins reduce ASCVD events across the full spectrum of primary and secondary prevention and form the cornerstone of guideline-directed lipid management [3]. For more than a decade, the 2018 AHA/ACC/Multisociety Guideline on the Management of Blood Cholesterol formed the basis of guidance on the initiation of lipid-lowering therapy, directing clinicians to use the Pooled Cohort Equations (PCE) to estimate 10-year ASCVD risk, with statin therapy broadly recommended for those at intermediate or high risk [4,5]. However, the PCE has since been shown to systematically overestimate contemporary 10-year ASCVD risk by 40%–80%, prompting calls for a more accurate and inclusive risk estimation framework [6,7].

In 2023, the AHA introduced the Predicting Risk of Cardiovascular Disease EVENTs (PREVENT) equations as an updated risk estimation tool derived from >3 million contemporary U.S. adults [8]. PREVENT differs from the PCEs in several clinically important respects: it removes race as a predictor variable, reflecting the scientific consensus that observed racial differences in cardiovascular risk reflect social rather than biological determinants [9]; it broadens the assessment age range from 40 to 75 to 30–79 years; it adds cardiometabolic risk predictors absent from the PCEs, including body mass index (BMI), glycemic factors (HbA1c), kidney function parameters (eGFR and urinary albumin-to-creatinine ratio [UACR]), and utilizes zip code as a proxy for social deprivation; additionally, it offers both 10- and 30-year risk estimates. In March 2026, the ACC and AHA published the first comprehensive lipid guideline update in nearly eight years, formally replacing the 2018 guideline and adopting PREVENT-ASCVD as the preferred primary prevention risk assessment tool [10]. Beyond the risk model transition, the 2026 dyslipidemia guideline introduced substantively expanded statin indications (Fig. 1): a revised PREVENT-ASCVD–based risk threshold structure with intermediate risk considered at >5% (Class of Recommendation [COR] I); incorporation of 30-year risk to identify younger adults aged 30–59 years at low short-term but elevated lifetime ASCVD risk (COR IIa); new categorical statin indications for adults aged 40–75 years with chronic kidney disease (CKD, stage 3 or higher) or HIV infection, regardless of calculated LDL-C level (COR I); and elevated high-sensitivity C-reactive protein (hsCRP ≥2 mg/L to <10 mg/L) as a risk-enhancing factor to guide statin initiation in borderline-risk patients (COR IIa) [10].

**Fig. 1:**
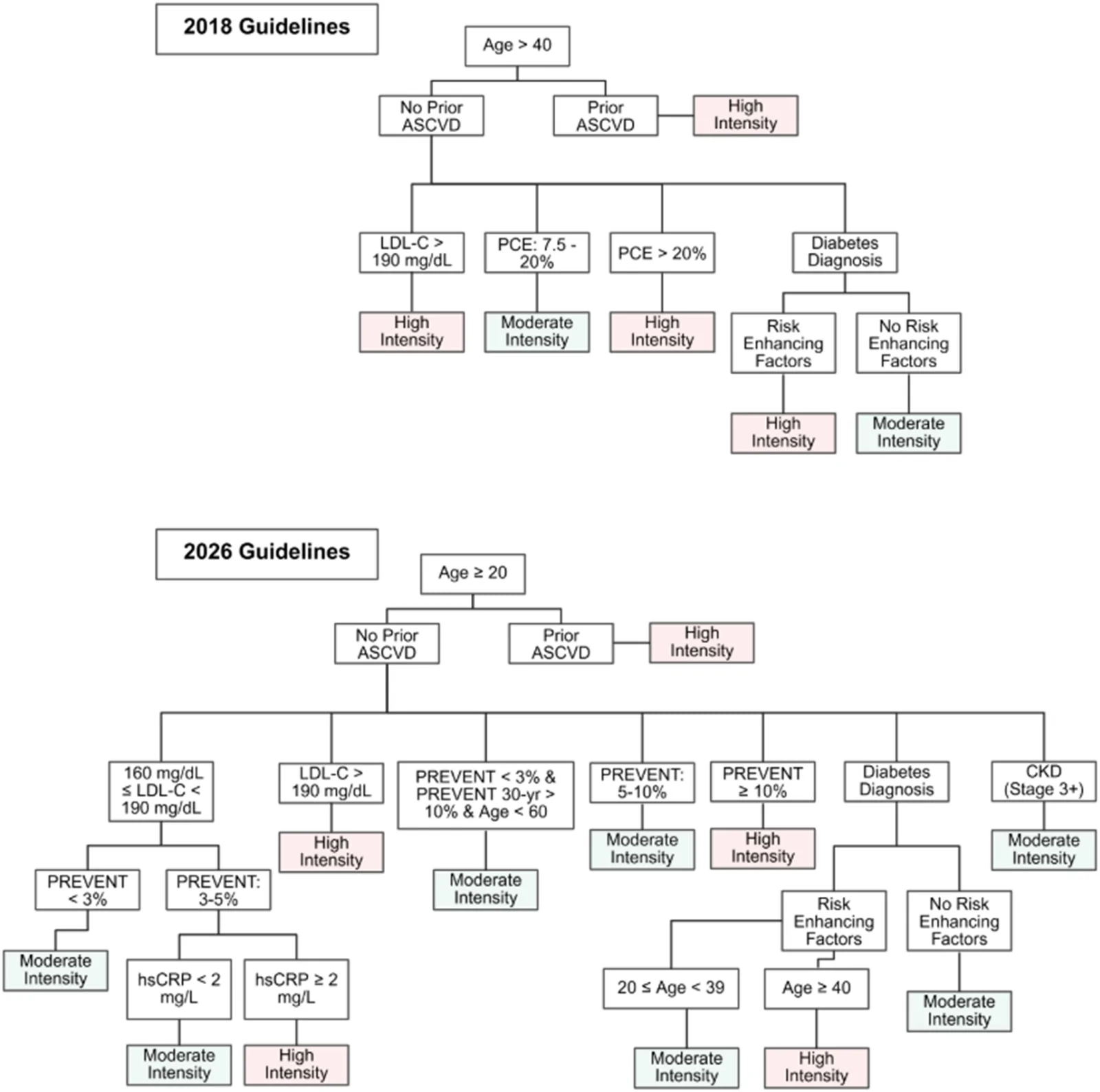
Guideline criteria for statin initiation.

Prior analyses applying PREVENT-ASCVD in isolation at the PCE-era threshold of ≥7.5%, without the accompanying changes to eligibility criteria embedded in the 2026 guideline or revision to risk thresholds, projected a net reduction of approximately 14.3 million statin-eligible U.S. adults, owing to PREVENT-ASCVD's systematically lower absolute risk estimates [6]. Whether this projected reduction would persist under the full 2026 guideline framework, with its refinement in risk estimation, broader eligibility criteria, and expanded age range, has not been evaluated in a nationally representative population. Our group previously demonstrated that the 2025 AHA/ACC High Blood Pressure Guideline, which adopted PREVENT-CVD at a recalibrated threshold within an otherwise similar eligibility structure, produced minimal net change in antihypertensive treatment eligibility [11]. Given the considerably more extensive structural changes in the 2026 dyslipidemia guideline, we hypothesized that its net impact on statin eligibility would diverge substantially from the hypertension experience. Using nationally representative data from the 2017–2020 National Health and Nutrition Examination Survey (NHANES), the present analysis sought to: (1) quantify the projected change in statin therapy eligibility under the 2026 compared with the 2018 guideline; (2) assess similarity and differences in eligibility across the two frameworks, including changes in recommended statin intensity; (3) quantify the independent contribution of each structural modification in the 2026 guideline to the observed expansion in eligibility; and (4) characterize the clinical profile of adults newly eligible under the 2026 guideline.

## 2 Methods

Data curation and analyses took place from March 2026 through April 2026. All analytical approaches and reporting contained herein were conducted in accordance with the Strengthening the Reporting of Observational Studies in Epidemiology (STROBE) guidelines.

### 2.1 Study cohort

This investigation used data from the 2017–2020 cycle of the National Health and Nutrition Examination Survey (NHANES), which began in 1999 and releases public-use health data from samples representative of the noninstitutionalized U.S. general population [12]. Individuals aged 30–79 years with available lipid profiles and complete data required for the 10-year Pooled Cohort Equations (PCE) and Predicting Risk of CVD EVENTs atherosclerotic cardiovascular disease (PREVENT-ASCVD) equations were included in the present analysis. Those with missing data, ages younger than 30 or older than 79, and those who were pregnant at the time of the survey were excluded from the analysis. The continuous NHANES study was reviewed and approved by the U.S. National Center for Health Statistics Institutional Review Board, and all participants provided informed written consent. This study was exempt from review at the local institutional review board due to the public and de-identified nature of the data.

### 2.2 Lipid concentrations and thresholds for treatment

Fasting serum samples were collected in the NHANES mobile examination centers and shipped to the University of Minnesota for analysis of serum lipid concentrations (total, low-density [LDL], and high-density [HDL] cholesterol and triglyceride). Starting with the 2017–2020 cycle and consistent with lipid guidelines recommendations, LDL was calculated using the Martin-Hopkins equation, [10,13] which was used in the primary analysis for the present work.

Eligibility for initiation of medical therapy was assessed according to the criteria outlined in the 20,18^5^ and 202,6^10^ AHA/ACC guidelines. These eligibility criteria are outlined in flowcharts depicted in Fig. 1. In addition, as noted in the 2026 ACC/AHA guidelines, high-sensitivity C-reactive protein (hsCRP) and chronic kidney disease (CKD) are associated with CVD risk and recommended as risk enhancers. As such, individuals with a borderline 10-year ASCVD risk and elevated hsCRP (≥2 mg/L to <10 mg/L) were considered eligible for high-intensity statin therapy [14]. Additionally, those with stage 3 or higher CKD were considered eligible for high-intensity statin therapy. In accordance with recommendations, individuals who were on dialysis and not already on a statin were not eligible for statin initiation.

Current use of lipid-lowering medication was defined as self-reported use of statins, as detailed in the medical prescription questionnaire in NHANES. Individuals already receiving statins were modeled as untreated by adjusting their LDL-C to reflect the expected changes from statin use, as has been done previously [6]. The effect on LDL-C was modeled as -30% for statins, and the pre-treatment LDL-C levels were estimated as (on-treatment LDL-C) / (0.70).

### 2.3 CVD risk prediction calculators

The history of clinical ASCVD was defined by self-report of prior physician-diagnosed coronary heart disease, myocardial infarction, or stroke. The calculation of PREVENT and PCE predicted ASCVD risk was performed as previously described [11,15,16]. Detailed, structured interviews were used to obtain relevant demographic information, including age at NHANES screening, race, ethnicity, and other sociodemographic characteristics. Systolic and diastolic arterial blood pressures were measured during standardized physical examinations in triplicate at 30-second intervals after a 5-minute seated rest period, and the mean was calculated. Hypertension was defined as a systolic blood pressure ≥ 140 mmHg, diastolic ≥ 90 mmHg, self-reported physician diagnosis of hypertension, or the use of antihypertensive medication. Serum measurements of creatinine, glucose, total cholesterol, high-density lipoprotein cholesterol, and hsCRP were performed at a central laboratory. Estimated glomerular filtration rate (eGFR) was calculated using the Chronic Kidney Disease Epidemiology Collaboration 2021 creatinine equation [17,18]. Diabetes mellitus was defined using the 2014 criteria established by the American Diabetes Association [19]: i.e., a fasting plasma glucose ≥126 mg/dL, an HbA1c ≥ 6.5% and/or use of insulin or anti-hyperglycemic medications. Chronic kidney disease (CKD) stage was defined according to the Kidney Disease: Improving Global Outcomes criteria as an eGFR<60 mL/min per 1.73 m^2^ or albuminuria, defined as urine albumin–creatinine ratio >30 mg/g [20]. Smoking status was determined as ‘currently’ or ‘never’, based on self-reported answers to risk behavior questionnaires.

### 2.4 Statistical analysis

To account for the complex survey design of the NHANES study, all analyses herein were adjusted for sample weights, clustering and stratification (determined in concordance with NHANES guidelines). Continuous data are presented as mean, and standard deviations (SD) or 95% confidence interval (95% CI), and categorical data are presented as count and percentage, unless otherwise noted. All statistical procedures were run in STATA version 19.5 (STATA Corp LLC) with statistical significance set *a priori* at α=0.05.

#### 2.4.1 Primary analysis

The primary objective of the current analyses was to compare the number of participants eligible for statin initiation under the 2018 lipid guidelines versus the new 2026 lipid guidelines. Participants were first stratified into 4 categories: not eligible under either set of guidelines, eligible only under the 2018 guidelines, eligible only under the 2026 guidelines, and eligible under both sets of guidelines. Sociodemographic and clinical characteristics were described for both the overall cohort and stratified across the categories of statin eligibility. The projected differences in statin eligibility between the 2018 and 2026 guidelines were estimated in the overall cohort and subsequently separated by age group, sex, race/ethnicity, health insurance status, and tertiles of family poverty-index ratio. Additionally, the percent change from 2018 to 2026 in eligibility was estimated and reported. Comparisons on statin eligibility were then made between individuals with who were eligible or not eligible under both guidelines or were eligible only under the 2018 or the 2026 guidelines, including any changes between moderate-dose and high-dose statin recommendations. These analyses were also performed after excluding all participants with prior ASCVD to focus solely on changes in statin eligibility in a primary-prevention population.

#### 2.4.2 Secondary analyses

To specifically evaluate factors associated with newly meeting eligibility criteria in 2026, individuals already eligible for statin therapy under the 2018 guidelines were excluded from these analyses. Univariate logistic regression was used to evaluate the characteristics associated with new eligibility under the 2026 guidelines, with age, sex, BMI, total cholesterol, eGFR, smoking status, hypertension, the use of anti-hypertensive mediation, race/ethnicity, education level, family poverty-to-income ratio were entered as model covariates. Diabetes mellitus was not included as a predictor given its role as an indication for statin initiation under the 2018 guidelines. Results are reported as odds ratios (ORs) with corresponding 95% confidence intervals (CIs). Because several covariates (e.g., eGFR, total cholesterol) are themselves components of the 2026 eligibility criteria, these models are intended to describe the clinical profile of newly eligible adults rather than to identify independent causal predictors; the independent contribution of each guideline modification is quantified separately in the sequential removal analysis.

Additionally, the contributing roles of specific guideline modifications to new eligibility were investigated. These modifications were: 1) the lower age threshold, 2) the substitution of PCE-derived risk for the PREVENT calculator, 3) the lower LDL threshold, 4) the inclusion of CKD for eligibility, 5) the inclusion of CRP as a risk enhancer, and 6) the incorporation of 30-year risk estimates for younger individuals at borderline risk. To determine the impact each of these revisions had on new eligibility, the 2026 eligibility criteria were re-applied to the newly eligible population with each modification removed or reversed individually. This allowed for the determination of the percent change in eligibility and 95% confidence associated with each new 2026 guideline criterion.

#### 2.4.3 Sensitivity analyses

A series of sensitivity analyses were conducted to evaluate the robustness of present analytical approaches. Given the different methods of estimating LDL-C the above analyses were reanalyzed using both the Friedewald and National Institutes of Health (NIH) LDL-C calculations. Total cholesterol, HDL cholesterol, and triglycerides from the NHANES data were used to recalculate LDL-C using the Friedewald and National Institutes of Health equations. Sensitivity analyses also included replacing the base PREVENT 10-yr and 30-yr models with three expanded PREVENT models: PREVENT + HbA1c, PREVENT + urinary albumin-creatinine ratio (UACR), and the full PREVENT model, which includes HbA1c, UACR, and the social deprivation index. The social deprivation index is not available in NHANES public data, therefore the full PREVENT model was calculated for individuals with the social deprivation index set to “missing”. To determine the reliability of our approach to estimating pre-treatment levels of LDL in individuals already taking statins, sensitivity analyses included models where individuals already on a statin were removed instead of modified.

## 3 Results

### 3.1 Eligibility for lipid-lowering therapy

A total of 2747 individuals were included in analyses, representative of 77,765,846 individuals after adjusting for the survey-weighted design of NHANES. The cohort had a mean age of 52.0 years (95% CI: 50.7–53.3 years) and balanced distribution by sex (50.6% women). Of these, 28.2% and 16.5% reported a history of hypertension and diabetes mellitus. 9.0% had prior ASVD (Table 1). Based on the PCE risk calculator thresholds used in the 2018 guidelines for initiating lipid-lowering medication, 9.7%, 23.0%, 12.2%, and 55.0% were categorized as high, intermediate, borderline, or low risk (**Supplemental Table 1**). In contrast, using the PREVENT-ASCVD risk calculator implemented in the 2026 guidelines, the corresponding proportions were 9.4%, 19.2%, 14.6%, and 56.8%, respectively.

**Table 1:** Overall cohort characteristics.

Unweighted Sample Size	2 747
Weighted Sample Size	77 765 846
Age, yrs	52.0 (50.7–53.3)
Women, %	50.6 (47.6–53.7)
*Sociodemographic Characteristics*	
Race/Ethnicity	
Mexican American, %	7.7 (6.0–10.0)
Non-Hispanic Asian, %	5.6 (4.0–7.8)
Non-Hispanic Black, %	10.3 (7.7–13.7)
Non-Hispanic White, %	64.8 (59.9–69.4)
Other Hispanic, %	7.0 (5.5–8.9)
Other/Multiracial, %	4.6 (3.5–6.0)
Education Level	
High School Graduate or Less, %	36.5 (33.2–40.0)
Some College or AA Degree, %	29.0 (25.1–33.3)
College Graduate or Above, %	34.4 (29.7–39.5)
No Health Insurance, %	11.8 (9.5–14.6)
Currently Unemployed, %	34.5 (31.5–37.6)
Family PIR, a.u.	3.3 (3.1–3.4)
*Clinical Measures*	
Weight, kg	85.8 (84.1–87.5)
Height, cm	168.7 (168.1–169.3)
Body Mass Index, kg/m2	30.1 (29.6–30.5)
SBP, mmHg	122.4 (121.4–123.5)
DBP, mmHg	75.2 (74.4–76)
Total Cholesterol, mg/dL	190.1 (186.8–193.4)
Triglycerides, mg/dL	111.2 (106.7–115.8)
HDL, mg/dL	54.2 (52.8–55.6)
Non-HDL-c, mg/dL	135.9 (132.5–139.3)
HbA1c, %	5.8 (5.7–5.8)
eGFR, mL/min/1.73m2	94 (92.4–95.6)
UACR, a.u.	27.5 (21.8–33.1)
hsCRP, mg/L	3.9 (3.4–4.3)
PREVENT 10-yr, %	3.7 (3.4–4.1)
PREVENT 30-yr, %	12.7 (12–13.4)
Current Smoker, %	17.4 (15–20.2)
Hypertension, %	28.2 (25.8–30.8)
Antihypertensives, %	12.8 (11.4–14.2)
Diabetes Mellitus, %	16.5 (14.9–18.2)
Antihyperglycemics, %	3.2 (2.4–4.2)
Prior ASCVD, %	9.0 (7.2–11.4)

Differences in eligibility between the two guidelines are illustrated in Fig. 2. Eligibility for statin therapy was 39.8% (95% CI: 36.5–43.3), representing 31 million individuals, according to the 2018 guidelines. Conversely, under the 2026 guidelines, a total of 42.2 million individuals (95% CI: 39.5–47.3), or 54.3% (95% CI: 51.2–57.3) of the cohort, were eligible for statin initiation. This reflected a 36.2% (95% CI: 28.0–44.4) increase in overall eligibility between 2018 and 2026. A major driver of this increase occurred among younger individuals under 50 years of age, whose eligibility increased by 104.9% (95% CI: 66.5–143.3), representing an additional 3.8 million individuals. Similar absolute increases in eligibility were observed for men and women, with a greater relative change for women. In addition, under the 2026 guidelines, an additional 1.7 million (95% CI: 0.8–2.6) uninsured and 1.9 million (95% CI: 1.2–2.6) individuals with a low poverty-income ratio would become eligible to initiate statins. These findings indicate that the new 2026 guideline, guided by the PREVENT risk framework, broadens treatment recommendations across multiple groups.

**Fig. 2:**
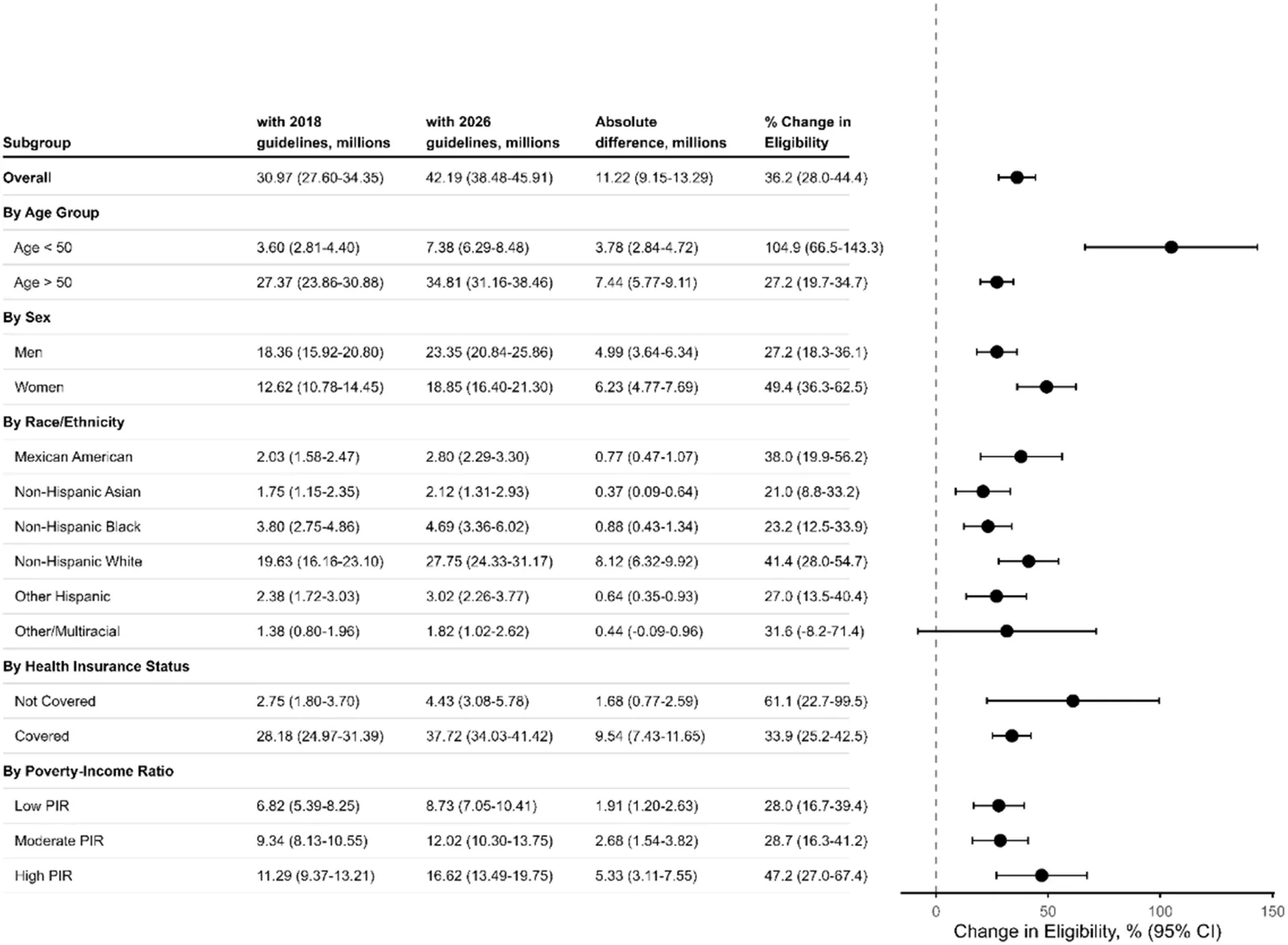
Differences in statin eligibility between the 2018 and the 2026 guidelines in the overall cohort and stratified by age, sex, race/ethnicity, health insurance status, and poverty-income ratio.

In the primary-prevention analysis, a total of 2747 individuals were included, representing 70.7 million individuals after adjusting for the survey-weighted design of NHANES. Notably, since the 2026 guidelines did not modify statin recommendations for individuals with prior ASCVD, the primary prevention subgroup analysis revealed an identical increase in the absolute number of individuals eligible for statin therapy (**Supplementary Figure 1**).

### 3.2 Interguideline changes in eligibility

Both guidelines recommended statin initiation for a large portion of adults (43.6%, 95% CI: 40.6–46.7), whereas approximately 16.5% (95% CI: 14.5–18.9) of adults were newly eligible only under the 2026 guidelines (Table 2). This group of newly eligible individuals was, on average, 52.5 years old (95% CI: 51.1–54.0) and was evenly distributed between men and women (51.3% women, 95% CI: 44.7–57.9). Self-reported uninsured individuals comprised a substantial portion, at 14.8% (95% CI: 9.4–22.6) uninsured, and 31.2% (95% CI: 23.2–40.6) were unemployed. The average family PIR was 3.4 (95% CI: 3.0–3.7). Further demographic characteristics and clinical profiles of the newly eligible individuals is presented in **Supplemental Table 2**. The number of adults who were only eligible under the 2018 guidelines and lost eligibility under the new guidelines was substantially smaller, representing 2.1% of adults (95% CI: 1.5–2.9). Fig. 3 illustrates the agreement and differences in treatment eligibility between the 2018 and 2026 guidelines. Fig. 4 outlines the changes in statin recommendations between the 2018 and 2026 guidelines, categorized by statin intensity. Notably, there was substantial agreement in the context of no statin recommendation (43.6%, 95% CI: 40.6–46.7) and with high-dose statins (25.0%, 95% CI: 22.3–27.8). There was a 13.7% (95% CI: 11.8–15.8) increase in transition from no statin recommendation under the 2018 guidelines to moderate-dose statin recommendations under the 2026 guidelines. A smaller proportion of individuals intensified treatment from moderate to high dose under the 2026 guidelines (1.7%, 95% CI: 1.3–2.4). The demographic characteristics and clinical profiles of the newly eligible individuals for primary prevention are presented in **Supplemental Table 3**.

**Table 2:** Characteristics of groups according to eligibility based on recommendations from 2018 to 2026 AHA/ACC thresholds for initiation of statin treatment.

	2018 and 2026 Similar Eligibility		2018 and 2026 Differing Eligibility	
Primary Analysis	Not Eligible with 2018 or 2026	Eligible with both 2018 and 2026	Eligible with 2018 Only	Eligible with 2026 Only
Unweighted Sample Size	1012	1296	80	359
Weighted Sample Size, millions	33.9	29.3	1.6	12.9
Age, yrs	43.0 (41.8–44.2)	62.2 (61.1–63.3)	60.0 (58.3–61.6)	52.5 (51.1–54.0)
Women, %	59.5 (53.2–65.5)	41.7 (37.4–46.1)	22.5 (11.0–40.6)	51.3 (44.7–57.9)
				
*Sociodemographic Characteristics*				
Race/Ethnicity				
Mexican American, %	9.2 (6.3–13.3)	6.6 (5.0–8.7)	5.8 (3.0–11.1)	6.7 (4.6–9.7)
Non-Hispanic Asian, %	6.1 (4.2–8.9)	5.4 (3.9–7.5)	9.4 (3.9–20.9)	4.1 (2.0–7.9)
Non-Hispanic Black, %	9.1 (6.4–12.7)	12.0 (8.9–16.0)	16.3 (8.4–29.3)	8.9 (6.0–13.1)
Non-Hispanic White, %	63.9 (56.9–70.3)	63.8 (57.8–69.5)	56.6 (35.9–75.2)	70.4 (64.4–75.7)
Other Hispanic, %	6.9 (4.8–9.7)	7.8 (5.9–10.1)	6.4 (2.2–17.1)	5.8 (3.7–8.9)
Other/Multiracial, %	4.9 (3.2–7.5)	4.4 (3.0–6.4)	5.4 (0.9–27.5)	4.1 (1.7–9.8)
Education Level				
High School Graduate or Less, %	28.1 (23.9–32.7)	46.4 (42.4–50.5)	42.6 (22.7–65.2)	35.6 (27.1–45.2)
Some College or AA Degree, %	31.9 (26.8–37.6)	26.5 (22.6–30.9)	33.2 (18.0–53.0)	26.7 (19.2–35.9)
College Graduate or Above, %	40.0 (34.3–46.0)	27.1 (22.0–32.9)	24.2 (12.7–41.3)	37.6 (28.2–48.1)
No Health Insurance, %	13.4 (10.3–17.2)	8.6 (6.0–12.1)	13.9 (4.4–36.3)	14.8 (9.4–22.6)
Currently Unemployed, %	19.2 (16.8–22.0)	53.3 (49.2–57.4)	38.8 (23.1–57.3)	31.2 (23.2–40.6)
Family PIR, a.u.	3.4 (3.2–3.5)	3.1 (2.9–3.2)	2.8 (2.2–3.3)	3.4 (3.0–3.7)
				
*Clinical Measures*				
Weight, kg	83.2 (80.4–85.9)	88.5 (87.1–90.0)	77.0 (73.2–80.8)	87.9 (84.6–91.2)
Height, cm	168.7 (167.6–169.8)	168.3 (167.4–169.2)	169.2 (166.0–172.4)	169.7 (168.1–171.2)
Body Mass Index, kg/m2	29.1 (28.3–29.9)	31.2 (30.6–31.8)	26.9 (25.7–28.0)	30.5 (29.6–31.4)
SBP, mmHg	115.0 (113.6–116.4)	130.0 (128.4–131.6)	126.9 (122.7–131.2)	125.0 (122.4–127.6)
DBP, mmHg	73.5 (72.3–74.6)	75.7 (74.6–76.8)	76.1 (73.8–78.5)	78.5 (77.2–79.8)
Total Cholesterol, mg/dL	186.2 (182.7–189.8)	189.9 (183.1–196.7)	197.4 (186.5–208.3)	200.4 (194.7–206.0)
Triglycerides, mg/dL	93.9 (89.5–98.3)	127.6 (118.5–136.6)	108.1 (62.4–153.9)	122.0 (111.9–132.1)
HDL, mg/dL	57.5 (55.7–59.3)	51.2 (49.6–52.8)	55.9 (50.4–61.4)	51.8 (49.1–54.5)
Non-HDL-c, mg/dL	128.8 (124.8–132.7)	138.6 (132.1–145.2)	141.5 (129.2–153.8)	148.5 (143.1–154.0)
HbA1c, %	5.3 (5.3–5.4)	6.3 (6.1–6.4)	5.6 (5.5–5.7)	5.7 (5.6–5.8)
eGFR, mL/min/1.73m2	101.2 (99.3–103.0)	85.9 (83.6–88.2)	94.2 (89.3–99.1)	92.9 (90.9–94.9)
UACR, a.u.	17.7 (7.1–28.2)	46.5 (31.6–61.3)	8.5 (4.8–12.3)	13.5 (9.3–17.7)
hsCRP, mg/L	3.4 (2.8–4.0)	4.3 (3.7–4.9)	1.6 (0.8–2.3)	4.4 (3.1–5.8)
PREVENT 10-yr, %	0.0 (0.0–0.2)	48.6 (45.3–51.9)	0.0 (0.0–0.0)	5.2 (3.0–8.8)
PREVENT 30-yr, %	92.5 (89.0–95.0)	12.1 (9.5–15.4)	1.5 (0.4–5.6)	71.5 (62.7–79.0)
Current Smoker, %	14.8 (11.9–18.2)	18.5 (15.3–22.2)	27.3 (16.1–42.4)	20.8 (16.2–26.2)
Hypertension, %	12.9 (10.3–15.9)	44.8 (41.2–48.4)	25.4 (14.3–41.2)	31.1 (23.5–39.8)
Antihypertensives, %	4.7 (3.6–6.1)	22.5 (20.1–25.1)	15.3 (7.1–29.9)	11.5 (6.8–18.9)
Diabetes Mellitus, %	0.1 (0.1–0.3)	40.9 (37.1–44.8)	0.0 (0.0–0.0)	5.9 (3.0–11.3)
Antihyperglycemics, %	0.0 (0.0–0.2)	7.3 (5.4–9.7)	0.0 (0.0–0.0)	2.6 (0.7–8.9)
Prior ASCVD, %	0.0 (0.0–0.0)	23.9 (19.8–28.4)	0.0 (0.0–0.0)	0.0 (0.0–0.0)

Continuous variables are reported as mean and 95% confident interval.

Categorical variables are reported as percentage and 95% confidence interval.

**Fig. 3:**
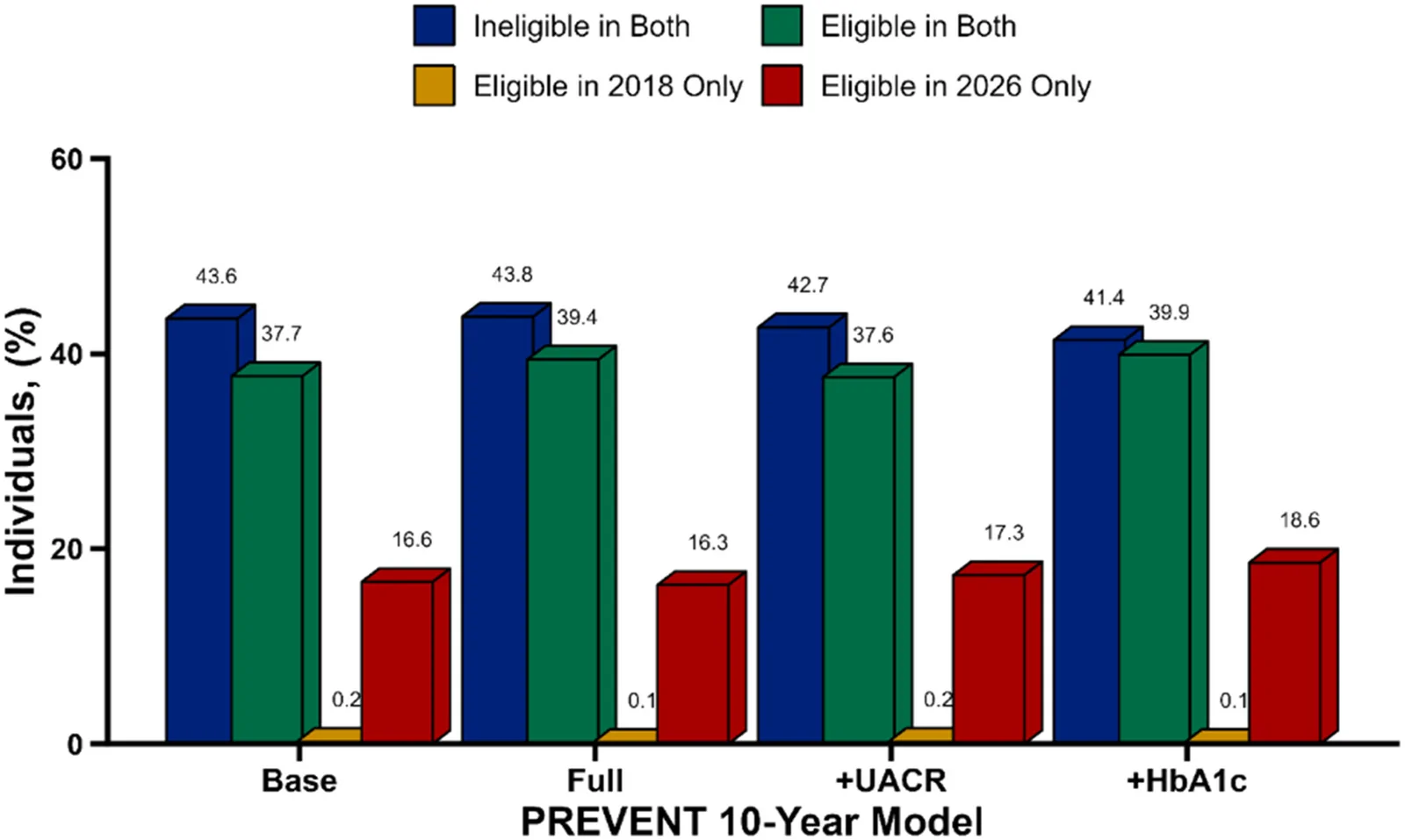
The proportion of individuals in each category: not eligible under 2018 or 2026 guidelines, eligible for statin initiation under both guidelines, eligible solely under the 2018 guidelines, and eligible solely under the 2026 guidelines.

**Fig. 4:**
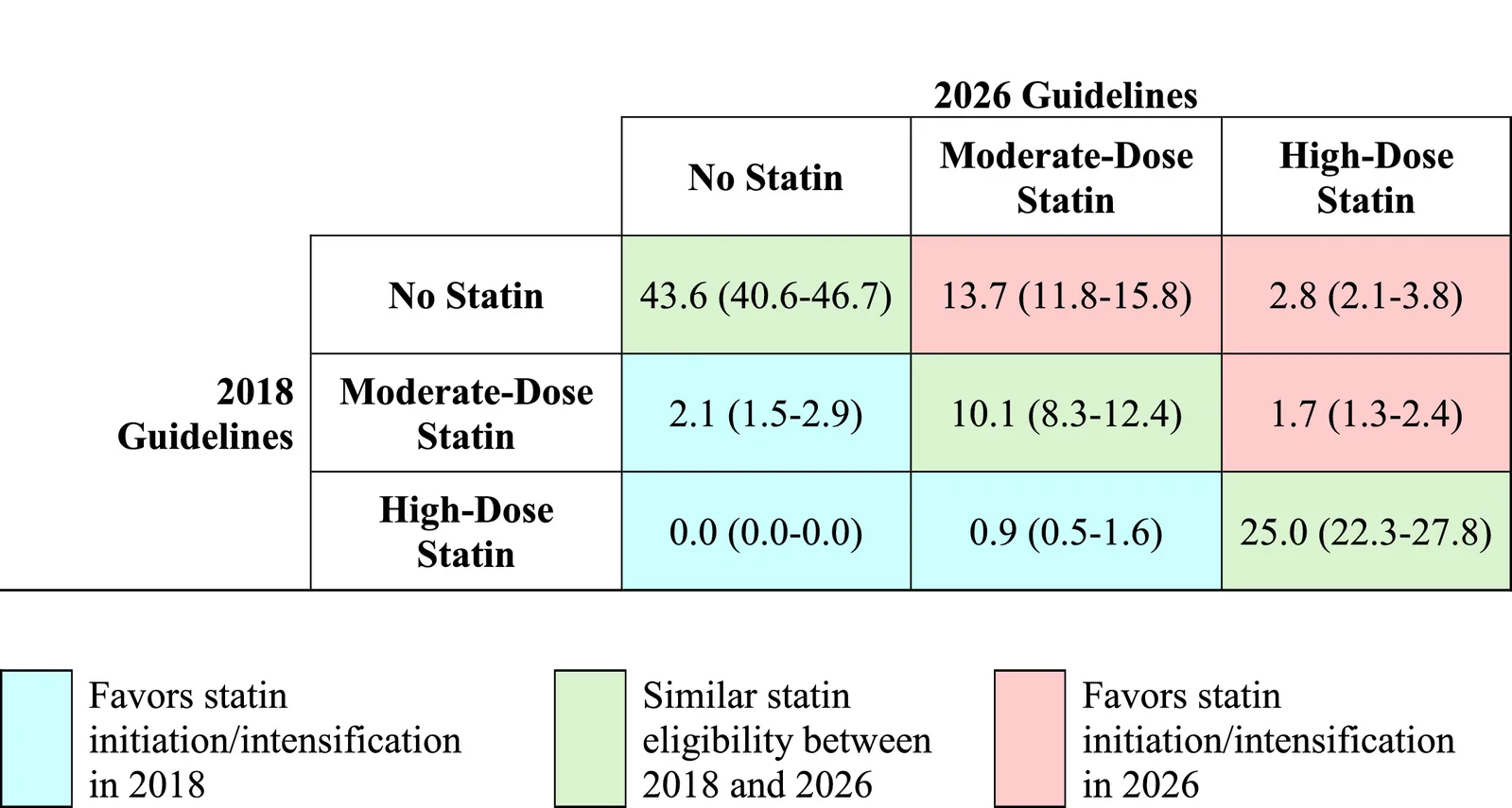
Agreement and disagreement in statin therapy recommendations between the 2018 and 2026 guidelines according to statin intensity. Values within each cell represent the percentage of the overall cohort (95% confidence interval). Green cells indicate agreement between the 2018 and 2026 guidelines, whereas blue and red cells indicate disagreement favoring greater statin initiation or intensification under the 2018 and 2026 guidelines, respectively.

### 3.3 Predictors and contributors to new eligibility under the 2026 guidelines

Fig. 5 illustrates the predictors of new eligibility under the 2026 guidelines. Among individuals not previously eligible for statin therapy under the 2018 guidelines, greater age, greater body mass index, higher total cholesterol, lower estimated glomerular filtration rate (eGFR), smoking, and the presence of hypertension were each associated with an increased likelihood of newly meeting statin eligibility criteria under the 2026 guidelines. Women were less likely to be in the newly eligible category compared to men. Conversely, race/ethnicity, education level, and family poverty-to-income ratio (PIR) were not significantly associated with new eligibility under the 2026 guidelines. Univariable and multivariable model results are shown in **Supplemental Tables 4–5.**

**Fig. 5:**
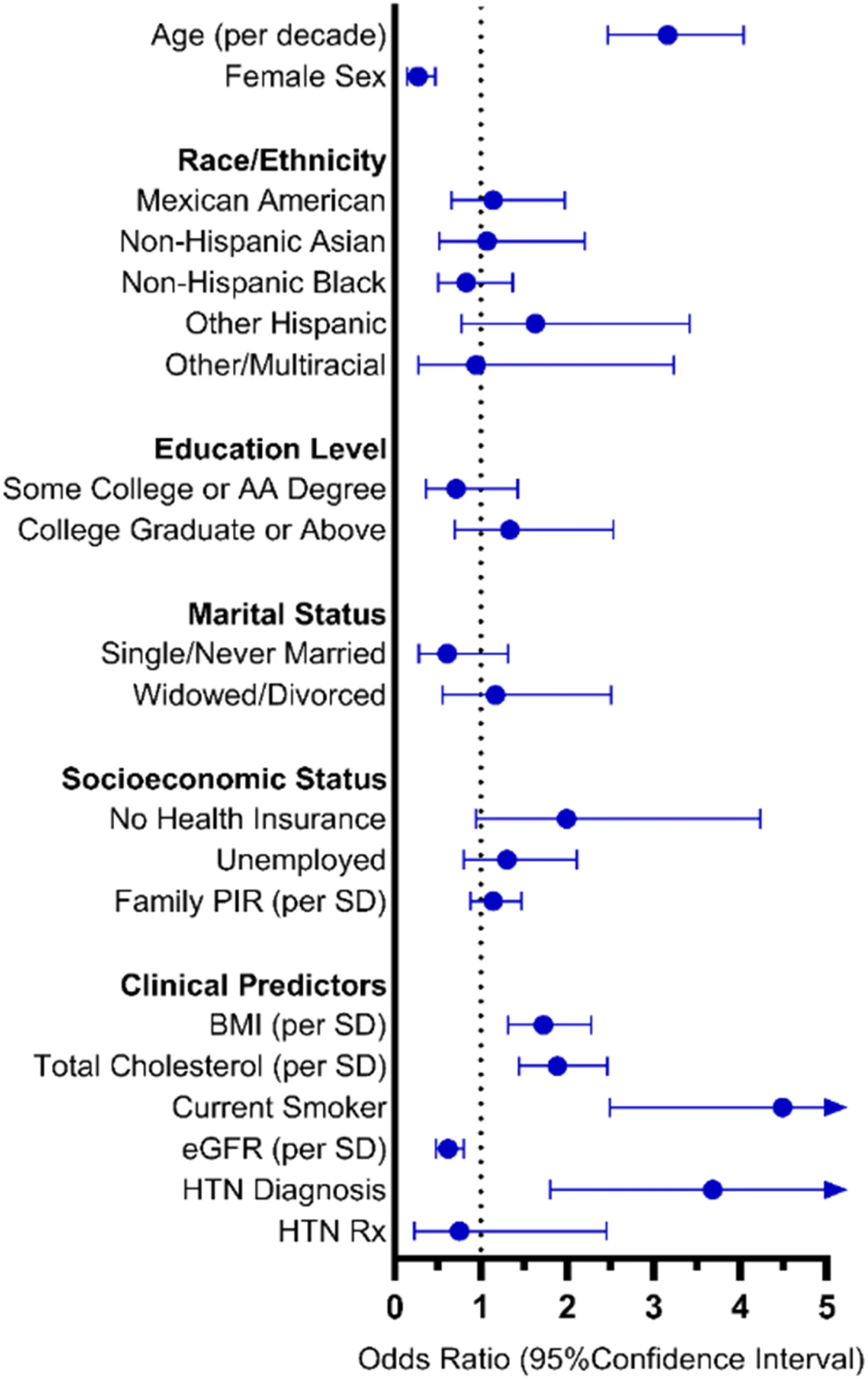
Multivariate logistic regression results characterizing the clinical profile of adults newly eligible under the 2026 lipid guidelines.

Sequential removal of each individual update to the 2026 guidelines for statin initiation revealed that multiple components contributed to at least 10% of the change in new eligibility. Specifically, the largest contributor to new eligibility was the 30-year risk prediction (66.1%, 95% CI: 60.6–71.7). The modified age threshold (9.7%, 95% CI: 5.5–14.0), use of PREVENT risk (10.8%, 95% CI: 9.4–12.2), modified LDL criteria (5.8, 95% CI: 1.0–10.6), and inclusion of CKD (0.2%, 95% CI: -0.1–0.5) and CRP (14.8%, 95% CI: 12.3–17.3) also had varying degrees of contributions to the increased number of eligible individuals (Fig. 6).

**Fig. 6:**
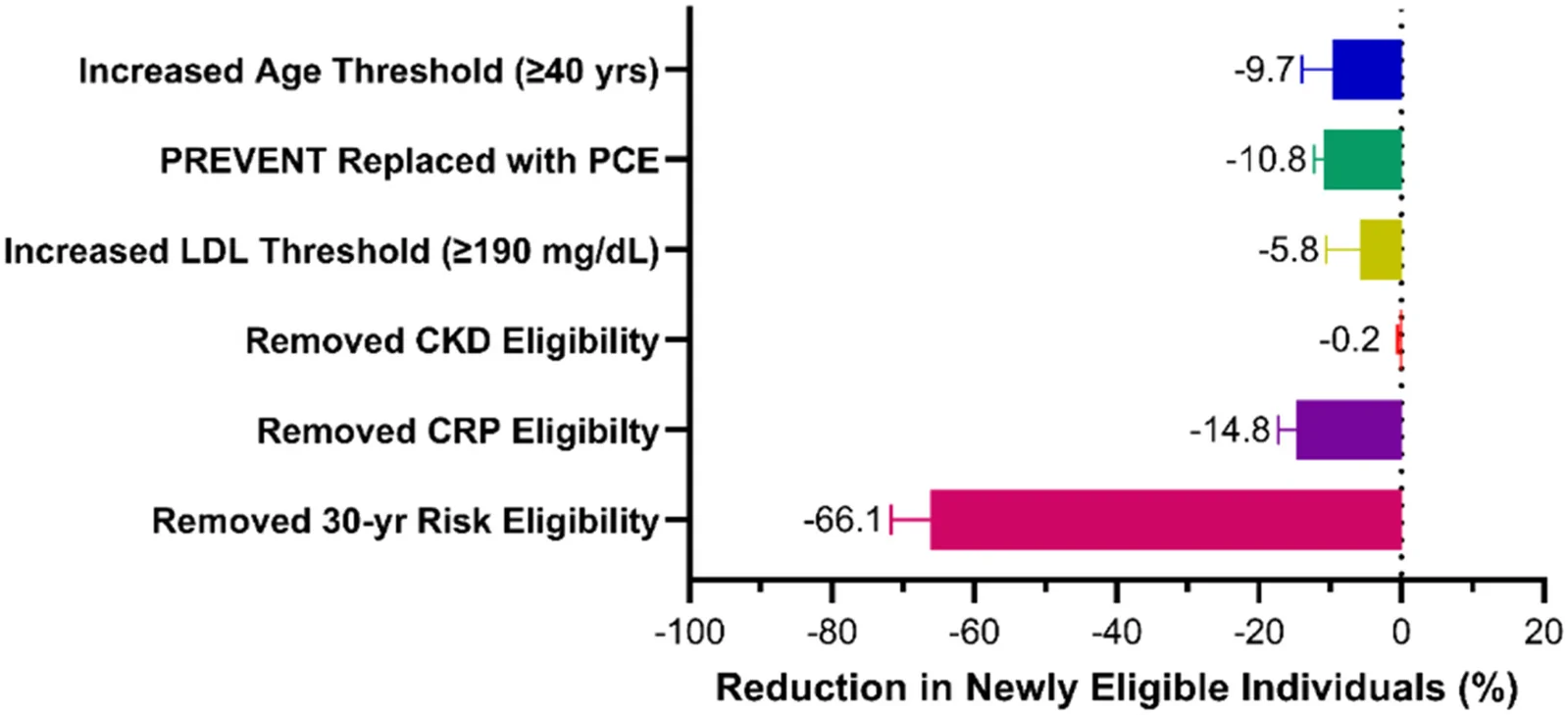
Percent-reductions in the newly eligible population after sequential removal or reversal of updates to the 2026 lipid guidelines.

### 3.4 Sensitivity analyses

Findings were not substantially altered when replacing the Martin-Hopkins-calculated LDL-C values with values calculated using either the NIH equation 2 or the Friedewald equation (**Supplemental Table 6–7**). Similarly, replacing the base 10-year and 30-year PREVENT calculations with PREVENT+HbA1c (**Supplemental Table 8**), PREVENT+UACR (**Supplemental Table 9**), or the full PREVENT calculator (**Supplemental Table 10**) did not grossly alter findings. Interestingly, inclusion of HbA1c broadened the eligibility of the 2026 guidelines, which may reflect the ability to include those with pre-diabetes even before a clinical diabetes diagnosis. Finally, removing individuals currently on statins, rather than estimating their pre-treatment statin levels, did not notably change the results (**Supplemental Table 11**).

## 4 Discussion

In this nationally representative analysis of U.S. adults aged 30–79 years, the 2026 ACC/AHA Dyslipidemia Guideline substantially expanded statin therapy eligibility compared with the 2018 cholesterol guideline. More than half of adults in this age range met criteria for statin initiation under the updated framework, with an estimated 11.2 million additional adults becoming eligible. Importantly, this expansion did not occur by displacing prior recommendations. Most adults were classified consistently across both frameworks, high-intensity statin recommendations were largely preserved among those at higher established risk, and only a small proportion of adults lost eligibility. These findings suggest that the 2026 guideline extends preventive statin eligibility to additional risk groups while maintaining treatment recommendations for adults already identified as high risk under the prior guideline.

The net expansion in eligibility reflects multiple structural changes in the 2026 guideline that extend well beyond the substitution of PREVENT-ASCVD for the PCEs [4]. When PREVENT-ASCVD has been applied in isolation at the prior PCE-era threshold of ≥7.5%, prior analyses projected a net reduction of approximately 14.3 million statin-eligible U.S. adults owing to PREVENT-ASCVD's systematically lower absolute risk estimates [6]. The present findings demonstrate that under the full 2026 guideline framework, this projected reduction is not only reversed but substantially exceeded, confirming that the population-level impact of the guideline is driven by both the expanded eligibility criteria and revision of risk thresholds. This stands in contrast to the minimal net change in antihypertensive eligibility when the analogous PREVENT-CVD–based 2025 hypertension guideline was adopted without comparable structural expansion in eligibility criteria [11,21]. This underscores that the impact of PREVENT-based guidelines depends on the breadth of accompanying eligibility changes rather than the risk model alone.

A major driver of expanded eligibility was the incorporation of 30-year PREVENT-ASCVD risk estimation for adults aged 30–59 years, along with formal risk assessment beginning at age 30 [8]. This change is clinically meaningful because atherosclerosis develops over decades, and cumulative exposure to elevated LDL-C is a major determinant of later cardiovascular risk [3]. Under the prior framework, many younger adults with unfavorable lipid profiles or cardiometabolic risk factors could remain below 10-year treatment thresholds despite elevated longer-term risk. The updated guideline addresses this gap by allowing earlier identification of adults whose short-term risk appears low but whose cumulative lifetime exposure may justify preventive therapy. This shift is especially relevant for primary prevention, where the greatest opportunity lies in intervening before advanced disease develops. Beyond age expansion, the 2026 guideline introduced new categorical statin indications for CKD (stage 3+), reflecting robust evidence that CKD confers ASCVD risk independent of traditional risk factors, [22] and for borderline-risk adults with hsCRP ≥2 mg/L to <10 mg/L, grounded in evidence that elevated hsCRP identifies residual inflammatory cardiovascular risk beyond lipid-based models [23,24]. The present cohort's mean hsCRP of 4.4 mg/L in the newly eligible group suggests that inflammation-mediated reclassification was operative in a substantial proportion of these adults. The inability to ascertain HIV status in NHANES precluded modeling of the new categorical statin indication for persons living with HIV.

The pattern of similarities and differences in eligibility between the two guideline frameworks further supports the interpretation that the 2026 guideline extended rather than displaced the prior framework. Adults recommended for high-intensity statin therapy under the 2018 guideline generally remained eligible under the 2026 framework, while the largest shift occurred among adults moving from no statin recommendation to moderate-intensity statin therapy. Moderate-intensity statin therapy produces clinically meaningful LDL-C reductions and has demonstrated benefit in primary prevention with a favorable safety profile [10,25]. Thus, the updated guideline appears to broaden the reach of prevention without substantially weakening treatment recommendations for higher-risk adults [5]. Downward reclassification from moderate to no statin (2.1%) is consistent with the modest impact of PREVENT-ASCVD's lower risk estimates on those previously at the lower bound of intermediate PCE risk.

The clinical profile of adults newly eligible under the 2026 guideline also reflects the revised structure of the recommendations. Greater age, higher total cholesterol, lower eGFR, current smoking, hypertension, and antihypertensive medication use were each more prevalent among adults newly eligible under the 2026 guideline, while sex, race/ethnicity, and poverty-to-income ratio were similarly distributed between newly eligible and other adults. These differences in clinical characteristics are coherent with the structural changes of both PREVENT and the guideline that drive expanded eligibility. The enrichment of higher total cholesterol reflects both the new LDL-C ≥ 160 mg/dL statin indication and 30-year risk pathway for younger adults with persistently elevated LDL-C [10]. The higher prevalence of lower eGFR directly reflects the new categorical CKD indication [22]. The higher prevalence of hypertension and antihypertensive use reflect the elevated ASCVD risk burden carried by this group [26]. Notably, sensitivity analyses incorporating HgbA1c into the PREVENT-ASCVD model further broadened eligibility, likely by capturing individuals with pre-diabetes who are at higher risk [27]; this is a finding with implications for earlier identification of cardiometabolic risk.

The finding that new eligibility was not concentrated by race/ethnicity, education level, and poverty-to-income ratio should be interpreted with caution. These findings suggest that new eligibility was not concentrated within these demographic groups after accounting for modeled clinical factors, but they do not establish equitable implementation or treatment uptake. The shift to a race-agnostic PREVENT-ASCVD addresses longstanding concerns about the use of race in risk prediction, particularly given that observed racial differences in cardiovascular risk are shaped by social and structural conditions rather than biology [9,28]. However, removing race from prediction models may lower estimated risk for some Black adults, particularly if the model does not fully capture the cumulative effects of social adversity [6,29,30]. The 2026 guideline’s “Calculate, Personalize, Reclassify” framework, including optional refinement with SDI, HbA1c, UACR, and other risk-enhancing factors, may help address some of this concern when implemented carefully [10]. The equity implications of expanded eligibility will ultimately depend on whether newly eligible adults are reached, treated, and supported over time. Persistent differences in statin use by race, ethnicity, insurance status, and socioeconomic position suggest that guideline expansion alone is unlikely to close longstanding gaps in prescribing, affordability, adherence, or longitudinal lipid management without implementation strategies that improve access, support clinician uptake, strengthen shared decision-making, reduce medication cost barriers, and promote follow-up [[31], [32], [33]]. Without these parallel efforts, the potential public health benefit of the 2026 guideline may not reach the populations most likely to benefit from earlier prevention.

### 4.1 Limitations

Several limitations should be noted. First, the cross-sectional design using NHANES 2017–2020 estimates guideline eligibility rather than observed statin prescribing, initiation, adherence, or clinical outcomes, and secular changes since 2020 may affect contemporary estimates. Second, NHANES does not capture HIV serostatus, precluding assessment of the new HIV-related statin indication. Given the high prevalence of dyslipidemia among persons living with HIV, this limitation likely makes our estimates conservative. Third, NHANES does not capture Lp(a), CAC scoring, apolipoprotein B, or the full set of guideline risk-enhancing factors, each of which may influence statin eligibility and could further contribute to underestimation of true eligibility. Fourth, the social deprivation index required for the full PREVENT-ASCVD model is unavailable in public NHANES data, although sensitivity analyses setting SDI to missing did not materially alter findings. Fifth, pretreatment LDL-C among statin users was estimated using a standardized 30% back-calculation, which may not fully account for statin intensity, adherence, or individual response; however, sensitivity analyses excluding current statin users yielded consistent results. Sixth, NHANES includes only the noninstitutionalized civilian population, limiting generalizability to adults in long-term care, correctional settings, or other institutional environments. Finally, this analysis characterizes eligibility only and cannot determine whether expanded eligibility will translate into prescribing, medication access, adherence, or reductions in ASCVD events. Despite these limitations, this study has several strengths. NHANES provides a nationally representative, survey-weighted sample with standardized measurement of the clinical variables required to apply both the PCE and PREVENT-ASCVD frameworks. The analytic approach applied the full 2026 guideline eligibility criteria rather than modeling PREVENT-ASCVD in isolation, providing a more clinically relevant estimate of the guideline’s projected population-level impact. Findings were also robust across sensitivity analyses using multiple LDL-C calculation methods, expanded PREVENT model variants, alternative approaches to current statin users, and a primary prevention-only subgroup, supporting the consistency of the primary results.

**Figure:**
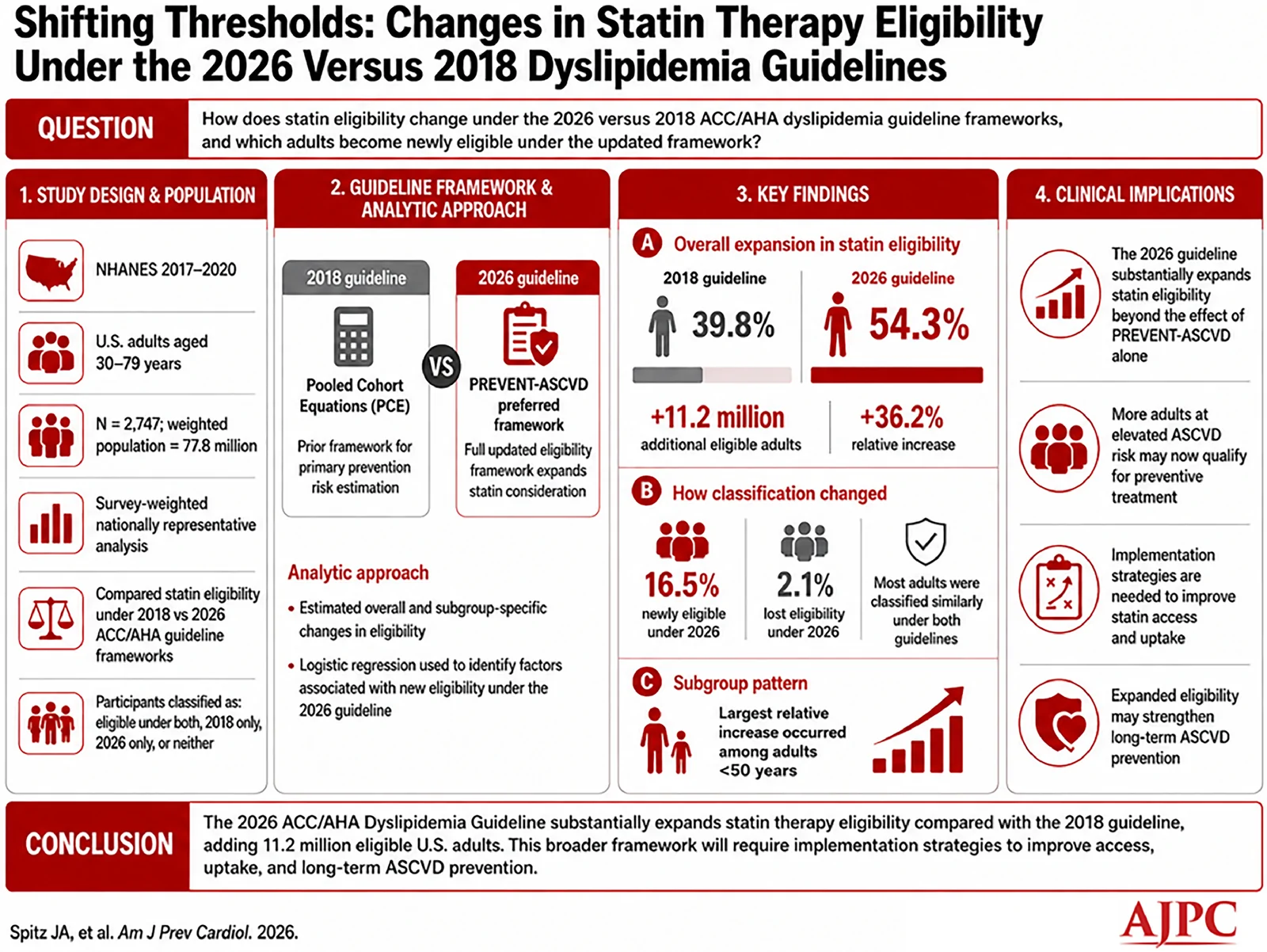

## 5 Conclusion

In a nationally representative sample of U.S. adults, the 2026 ACC/AHA Dyslipidemia guideline is projected to expand statin therapy eligibility by 36.2% compared with the 2018 cholesterol guideline, representing 11.2 million additional eligible individuals. This expansion is driven by risk assessment beginning at age 30, adoption of 30-year risk estimation, and new categorical indications including CKD, HIV, and elevated hsCRP. Importantly, the expansion occurred without displacing prior recommendations, with high concordance and largely preserved high-intensity statin recommendations. Eligibility expansion was also evenly distributed across race and sex. These findings underscore that the population-level impact of PREVENT-based guidelines depends on the full scope of accompanying eligibility criteria, not the risk model alone. Yet expanded eligibility does not ensure expanded treatment. Persistent racial, ethnic, and socioeconomic differences in statin prescribing mean the public health benefit of these guideline changes will require parallel investments in access, affordability, and broad implementation.

## Funding statement

No research funding was received for the conduct of this research and/or preparation of this manuscript.

## CRediT authorship contribution statement

**Jared A. Spitz:** Writing – review & editing, Writing – original draft, Conceptualization. **Faith E. Metlock:** Writing – review & editing, Writing – original draft, Visualization. **Bede Nriagu:** Writing – review & editing. **Carl Ade:** Writing – review & editing, Writing – original draft, Visualization, Methodology, Formal analysis. **Britton Scheuermann:** Writing – review & editing, Writing – original draft, Visualization, Formal analysis. **Lily Dastmalchi:** Writing – review & editing. **Garima Sharma:** Writing – review & editing, Conceptualization. **Samuel Kim:** Writing – review & editing.

## Declaration of competing interest

The authors declare that they have no known competing financial interests or personal relationships that could have appeared to influence the work reported in this paper.

## References

[1] Joynt Maddox K.E., Elkind M.S.V., Aparicio H.J., Commodore-Mensah Y., de Ferranti S.D., Dowd W.N., Hernandez A.F., Khavjou O., Michos E.D., Palaniappan L. (2024). Forecasting the burden of cardiovascular disease and stroke in the United States through 2050-prevalence of risk factors and disease: a presidential advisory from the American Heart Association. Circulation.

[2] Martin S.S., Aday A.W., Allen N.B., Almarzooq Z.I., Anderson C.A.M., Arora P., Avery C.L., Baker-Smith C.M., Bansal N., Beaton A.Z. (2025). 2025 Heart Disease and Stroke statistics: a report of US and global data from the American Heart Association. Circulation.

[3] Mihaylova B., Emberson J., Blackwell L., Keech A., Simes J., Barnes E.H., Voysey M., Gray A., Collins R., Baigent C. (2012). The effects of lowering LDL cholesterol with statin therapy in people at low risk of vascular disease: meta-analysis of individual data from 27 randomised trials. Lancet.

[4] Goff D.C., Lloyd-Jones D.M., Bennett G., Coady S., D'Agostino R.B., Gibbons R., Greenland P., Lackland D.T., Levy D., O'Donnell C.J. (2014). 2013 ACC/AHA guideline on the assessment of cardiovascular risk: a report of the American College of Cardiology/American Heart Association Task Force on Practice Guidelines. Circulation.

[5] Grundy S.M., Stone N.J., Bailey A.L., Beam C., Birtcher K.K., Blumenthal R.S., Braun L.T., de Ferranti S., Faiella-Tommasino J., Forman D.E. (2019). 2018 AHA/ACC/AACVPR/AAPA/ABC/ACPM/ADA/AGS/APhA/ASPC/NLA/PCNA Guideline on the Management of blood Cholesterol: a report of the American College of Cardiology/American Heart Association Task Force on Clinical Practice Guidelines. Circulation.

[6] Diao J.A., Shi I., Murthy V.L., Buckley T.A., Patel C.J., Pierson E., Yeh R.W., Kazi D.S., Wadhera R.K., Manrai A.K. (2024). Projected changes in statin and antihypertensive therapy eligibility with the AHA PREVENT cardiovascular risk equations. Jama.

[7] Yadlowsky S., Hayward R.A., Sussman J.B., McClelland R.L., Min Y.I., Basu S. (2018). Clinical implications of revised pooled cohort equations for estimating atherosclerotic cardiovascular disease risk. Ann Intern Med.

[8] Khan S.S., Matsushita K., Sang Y., Ballew S.H., Grams M.E., Surapaneni A., Blaha M.J., Carson A.P., Chang A.R., Ciemins E. (2024). Development and validation of the american heart association's prevent equations. Circulation.

[9] Ndumele C.E., Ferdinand K.C., Khan S.S. (2025). The role of race in cardiovascular disease risk prediction. Circulation.

[10] Members W.C., Blumenthal R.S., Morris P.B., Gaudino M., Johnson H.M., Anderson T.S., Bittner V.A., Blankstein R., Brewer L.C., Cho L. (2026). 2026 ACC/AHA/AACVPR/ABC/ACPM/ADA/AGS/APhA/ASPC/NLA/PCNA guideline on the management of dyslipidemia: a report of the american college of cardiology/american heart association joint committee on clinical practice guidelines. Circulation.

[11] Metlock F.E, Nriagu B.N., Scheuermann B., Ade C., Kwapong Y.A., Razavi A.C., Juraschek S., McCoy S.M., Dastmalchi L.N., Sharma G. (2026). Shifting thresholds: changes in antihypertensive eligibility under the 2025 versus 2017 hypertension guidelines. JACC Adv.

[12] Mirel L.B., Mohadjer L.K., Dohrmann S.M., Clark J., Burt V.L., Johnson C.L., Curtin L.R. (2013). National Health and Nutrition Examination Survey: estimation procedures, 2007-2010. Vital Health Stat.

[13] Martin S.S., Blaha M.J., Elshazly M.B., Toth P.P., Kwiterovich P.O., Blumenthal R.S., Jones S.R. (2013). Comparison of a novel method vs the Friedewald equation for estimating low-density lipoprotein cholesterol levels from the standard lipid profile. Jama.

[14] Pearson T.A., Mensah G.A., Alexander R.W., Anderson J.L., Cannon R.O., Criqui M., Fadl Y.Y., Fortmann S.P., Hong Y., Myers G.L. (2003). Markers of inflammation and cardiovascular disease: application to clinical and public health practice: a statement for healthcare professionals from the Centers for Disease Control and Prevention and the American Heart Association. Circulation.

[15] Goff D.C., Lloyd-Jones D.M., Bennett G., Coady S., D'Agostino R.B., Gibbons R., Greenland P., Lackland D.T., Levy D., O'Donnell C.J. (2014). 2013 ACC/AHA guideline on the assessment of cardiovascular risk: a report of the American College of Cardiology/American Heart Association Task Force on Practice Guidelines. J Am Coll Cardiol.

[16] Khan S.S., Matsushita K., Sang Y., Ballew S.H., Grams M.E., Surapaneni A., Blaha M.J., Carson A.P., Chang A.R., Ciemins E. (2024). Development and validation of the American Heart Association’s PREVENT equations. Circulation.

[17] Benetos A., Laurent S., Hoeks A.P., Boutouyrie P.H., Safar M.E. (1993). Arterial alterations with aging and high blood pressure. A noninvasive study of carotid and femoral arteries. Arterioscler Thromb.

[18] Levey A.S., Coresh J., Greene T., Marsh J., Stevens L.A., Kusek J.W., Van Lente F. (2007). Expressing the modification of diet in renal disease study equation for estimating glomerular filtration rate with standardized serum creatinine values. Clin Chem.

[19] (2010). Diagnosis and classification of diabetes mellitus. Diabetes Care.

[20] (2024). KDIGO 2024 clinical practice guideline for the evaluation and management of chronic kidney disease. Kidney Int.

[21] Khan S.S., Lloyd-Jones D.M., Abdalla M., Bello N.A., Blyler C.A., Carter J., Commodore-Mensah Y., Ferdinand K.C., Johnson H.M., Jones D. (2025). Use of risk assessment to guide decision-making for blood pressure management in the primary prevention of cardiovascular disease: a scientific statement from the American Heart Association and American College of Cardiology. JACC.

[22] Jankowski J., Floege J., Fliser D., Böhm M., Marx N. (2021). Cardiovascular disease in chronic kidney disease: pathophysiological insights and therapeutic options. Circulation.

[23] Ridker P.M., Danielson E., Fonseca F.A., Genest J., Gotto A.M., Kastelein J.J., Koenig W., Libby P., Lorenzatti A.J., MacFadyen J.G. (2008). Rosuvastatin to prevent vascular events in men and women with elevated C-reactive protein. N Engl J Med.

[24] Ridker P.M., Moorthy M.V., Cook N.R., Rifai N., Lee I.M., Buring J.E. (2024). Inflammation, cholesterol, lipoprotein(a), and 30-year cardiovascular outcomes in women. N Engl J Med.

[25] Kalra D.K., Ray K.K., Bajaj A., Kushner P.R., Wilcox M.L., Dicklin M.R., Kirkpatrick C.F., Maki K.C. (2026). Low-density lipoprotein cholesterol lowering and risk of major adverse cardiovascular events in primary prevention trials: a meta-analysis. J Clin Lipidol.

[26] Whelton S.P., McEvoy J.W., Shaw L., Psaty B.M., Lima J.A.C., Budoff M., Nasir K., Szklo M., Blumenthal R.S., Blaha M.J. (2020). Association of normal systolic blood pressure level with cardiovascular disease in the absence of risk factors. JAMA Cardiol.

[27] Neves J.S., Newman C., Bostrom J.A., Buysschaert M., Newman J.D., Medina J.L., Goldberg I.J., Bergman M. (2022). Management of dyslipidemia and atherosclerotic cardiovascular risk in prediabetes. Diabet Res Clin Pract.

[28] Powell-Wiley T.M., Baumer Y., Baah F.O., Baez A.S., Farmer N., Mahlobo C.T., Pita M.A., Potharaju K.A., Tamura K., Wallen G.R. (2022). Social determinants of cardiovascular disease. Circ Res.

[29] Razavi A.C., Kohli P., McGuire D.K., Martin S.S., Polonsky T.S., McEvoy J.W., Whelton S.P., Blumenthal R.S. (2024). PREVENT equations: a new era in cardiovascular disease risk assessment. Circ Cardiovasc Qual Outcomes.

[30] Metlock F.E., Stanislas K.A., Ogungbe O., Teshome B., Hinneh T., Turkson-Ocran R.-A., Himmelfarb C.R., Sharma G., Commodore-Mensah Y. (2026). Sex differences in the association between cumulative social risk and life’s essential 8 cardiovascular health scores in U.S. adults. Am J Preven Cardiol.

[31] Frank D.A., Johnson A.E., Hausmann L.R.M., Gellad W.F., Roberts E.T., Vajravelu R.K. (2023). Disparities in guideline-recommended statin use for prevention of atherosclerotic cardiovascular disease by race, ethnicity, and gender: a nationally representative cross-sectional analysis of adults in the United States. Ann Intern Med.

[32] Wong C., Marrast L., Rasul R., Srivastava R., Kuvin J., Roswell R., Conigliaro J., Kim E.J. (2024). Financial resources, access to care, and quality of care mediate racial disparities in statin usage for secondary prevention. PLoS One.

[33] Salami J.A., Warraich H., Valero-Elizondo J., Spatz E.S., Desai N.R., Rana J.S., Virani S.S., Blankstein R., Khera A., Blaha M.J. (2017). National trends in statin use and expenditures in the US adult population from 2002 to 2013: insights from the medical expenditure Panel Survey. JAMA Cardiol.

