# Supplementary material for: Changes in statin therapy eligibility under the 2026 versus 2018 dyslipidemia guidelines
Source: Am J Prev Cardiol. 2026 Jun 26;30:101719. doi: 10.1016/j.ajpc.2026.101719 (PMC13633900; doi:10.1016/j.ajpc.2026.101719)
Supplement: Supplementary file 1 [file mmc1.docx]

**SUPPLEMENTAL DOCUMENT**

**Supplementary Figure 1.** Differences in statin eligibility between the 2018 and the 2026 guidelines in the primary prevention analysis stratified by age, sex, race/ethnicity, health insurance status, and poverty-income ratio.

| **Supplemental Table 1.** Proportion of participants in each risk category for the PREVENT and Pooled Cohort Equations calculators. Data are presented as percent and 95% confidence interval. | |
| --- | --- |
| *PREVENT* |  |
| Low 10-yr Risk, % | 56.8 (52.9-60.6) |
| Borderline 10-yr Risk, % | 14.6 (12.6-17.0) |
| Intermediate 10-yr Risk, % | 19.2 (17.0-21.6) |
| High 10-yr Risk, % | 9.4 (7.9-11.2) |
|  |  |
| *Pooled Cohort Equations* |  |
| Low 10-yr Risk, % | 55.0 (51.3-58.6) |
| Borderline 10-yr Risk, % | 12.2 (10.4-14.4) |
| Intermediate 10-yr Risk, % | 23.0 (20.6-25.6) |
| High 10-yr Risk, % | 9.7 (8.2-11.6) |

| **Supplemental Table 2. Characteristics of the newly eligible cohort.** | |
| --- | --- |
| Unweighted Sample Size | 359 |
| Weighted Sample Size | 12.9 |
| Age, yrs | 52.5 (51.1-54.0) |
| Women, % | 51.3 (44.7-57.9) |
|  |  |
| *Sociodemographic Characteristics* |  |
| Race/Ethnicity |  |
| Mexican American, % | 6.7 (4.6-9.7) |
| Non-Hispanic Asian, % | 4.1 (2.0-7.9) |
| Non-Hispanic Black, % | 8.9 (6.0-13.1) |
| Non-Hispanic White, % | 70.4 (64.4-75.7) |
| Other Hispanic, % | 5.8 (3.7-8.9) |
| Other/Multiracial, % | 4.1 (1.7-9.8) |
| Education Level |  |
| High School Graduate or Less, % | 35.6 (27.1-45.2) |
| Some College or AA Degree, % | 26.7 (19.2-35.9) |
| College Graduate or Above, % | 37.6 (28.2-48.1) |
| No Health Insurance, % | 14.8 (9.4-22.6) |
| Currently Unemployed, % | 31.2 (23.2-40.6) |
| Family PIR, a.u. | 3.4 (3.0-3.7) |
|  |  |
| *Clinical Measures* |  |
| Weight, kg | 87.9 (84.6-91.2) |
| Height, cm | 169.7 (168.1-171.2) |
| Body Mass Index, kg/m2 | 30.5 (29.6-31.4) |
| SBP, mmHg | 125.0 (122.4-127.6) |
| DBP, mmHg | 78.5 (77.2-79.8) |
| Total Cholesterol, mg/dL | 200.4 (194.7-206.0) |
| Triglycerides, mg/dL | 122.0 (111.9-132.1) |
| HDL, mg/dL | 51.8 (49.1-54.5) |
| Non-HDL-c, mg/dL | 148.5 (143.1-154.0) |
| HbA1c, % | 5.7 (5.6-5.8) |
| eGFR, mL/min/1.73m2 | 92.9 (90.9-94.9) |
| UACR, a.u. | 13.5 (9.3-17.7) |
| hsCRP, mg/L | 4.4 (3.1-5.8) |
| PREVENT 10-yr, % | 5.2 (3.0-8.8) |
| PREVENT 30-yr, % | 71.5 (62.7-79.0) |
| Current Smoker, % | 20.8 (16.2-26.2) |
| Hypertension, % | 31.1 (23.5-39.8) |
| Antihypertensives, % | 11.5 (6.8-18.9) |
| Diabetes Mellitus, % | 5.9 (3.0-11.3) |
| Antihyperglycemics, % | 2.6 (0.7-8.9) |
| Prior ASCVD, % | 0.0 (0.0-0.0) |

| **Supplemental Table 3** | | | | |
| --- | --- | --- | --- | --- |
| **Sensitivity – Primary Prevention Only** | **Not Eligible with 2018 or 2026** | **Eligible with both 2018 and 2026** | **Eligible with 2018 Only** | **Eligible with 2026 Only** |
| Unweighted Sample Size | 1007 | 1004 | 80 | 359 |
| Weighted Sample Size, millions | 33.9 | 22.3 | 1.6 | 12.9 |
| Age, yrs | 43.0 (41.8-44.2) | 62.2 (60.9-63.5) | 60.0 (58.3-61.6) | 52.5 (51.1-54.0) |
| Women, % | 59.5 (53.2-65.5) | 41.2 (36.1-46.5) | 22.5 (11.0-40.6) | 51.3 (44.7-57.9) |
|  |  |  |  |  |
| *Sociodemographic Characteristics* |  |  |  |  |
| Race/Ethnicity |  |  |  |  |
| Mexican American, % | 9.2 (6.3-13.2) | 7.7 (5.8-10.3) | 5.8 (3.0-11.1) | 6.7 (4.6-9.7) |
| Non-Hispanic Asian, % | 6.1 (4.2-8.9) | 6.2 (4.4-8.6) | 9.4 (3.9-20.9) | 4.1 (2.0-7.9) |
| Non-Hispanic Black, % | 9.0 (6.4-12.6) | 11.9 (8.5-16.3) | 16.3 (8.4-29.3) | 8.9 (6.0-13.1) |
| Non-Hispanic White, % | 63.9 (57.0-70.4) | 62.0 (54.9-68.6) | 56.6 (35.9-75.2) | 70.4 (64.4-75.7) |
| Other Hispanic, % | 6.9 (4.8-9.7) | 8.8 (6.7-11.5) | 6.4 (2.2-17.1) | 5.8 (3.7-8.9) |
| Other/Multiracial, % | 4.9 (3.1-7.5) | 3.4 (2.2-5.3) | 5.4 (0.9-27.5) | 4.1 (1.7-9.8) |
| Education Level |  |  |  |  |
| High School Graduate or Less, % | 28.1 (23.9-32.7) | 45.2 (40.9-49.5) | 42.6 (22.7-65.2) | 35.6 (27.1-45.2) |
| Some College or AA Degree, % | 31.9 (26.8-37.5) | 26.6 (22.9-30.7) | 33.2 (18.0-53.0) | 26.7 (19.2-35.9) |
| College Graduate or Above, % | 40.0 (34.3-46.0) | 28.2 (23.4-33.7) | 24.2 (12.7-41.3) | 37.6 (28.2-48.1) |
| No Health Insurance, % | 13.4 (10.3-17.2) | 9.0 (6.1-13.0) | 13.9 (4.4-36.3) | 14.8 (9.4-22.6) |
| Currently Unemployed, % | 19.2 (16.7-21.9) | 52.1 (47.1-57.0) | 38.8 (23.1-57.3) | 31.2 (23.2-40.6) |
| Family PIR, a.u. | 3.4 (3.2-3.5) | 3.1 (2.9-3.3) | 2.8 (2.2-3.3) | 3.4 (3.0-3.7) |
|  |  |  |  |  |
| *Clinical Measures* |  |  |  |  |
| Weight, kg | 83.2 (80.4-85.9) | 88.2 (86.0-90.4) | 77.0 (73.2-80.8) | 87.9 (84.6-91.2) |
| Height, cm | 168.7 (167.6-169.8) | 168.3 (167.3-169.3) | 169.2 (166.0-172.4) | 169.7 (168.1-171.2) |
| Body Mass Index, kg/m2 | 29.1 (28.3-29.9) | 31.1 (30.2-32.0) | 26.9 (25.7-28.0) | 30.5 (29.6-31.4) |
| SBP, mmHg | 115.0 (113.6-116.4) | 129.8 (128.3-131.3) | 126.9 (122.7-131.2) | 125.0 (122.4-127.6) |
| DBP, mmHg | 73.5 (72.3-74.6) | 75.3 (74.3-76.4) | 76.1 (73.8-78.5) | 78.5 (77.2-79.8) |
| Total Cholesterol, mg/dL | 186.3 (182.7-189.8) | 194.6 (186.8-202.3) | 197.4 (186.5-208.3) | 200.4 (194.7-206.0) |
| Triglycerides, mg/dL | 93.9 (89.5-98.3) | 127.8 (118.4-137.3) | 108.1 (62.4-153.9) | 122.0 (111.9-132.1) |
| HDL, mg/dL | 57.5 (55.7-59.3) | 51.2 (49.7-52.8) | 55.9 (50.4-61.4) | 51.8 (49.1-54.5) |
| Non-HDL-c, mg/dL | 128.8 (124.8-132.7) | 143.3 (136.1-150.6) | 141.5 (129.2-153.8) | 148.5 (143.1-154.0) |
| HbA1c, % | 5.3 (5.3-5.4) | 6.3 (6.1-6.4) | 5.6 (5.5-5.7) | 5.7 (5.6-5.8) |
| eGFR, mL/min/1.73m2 | 101.2 (99.3-103.0) | 86.1 (83.8-88.4) | 94.2 (89.3-99.1) | 92.9 (90.9-94.9) |
| UACR, a.u. | 16.9 (6.4-27.4) | 40.5 (26.8-54.1) | 8.5 (4.8-12.3) | 13.5 (9.3-17.7) |
| hsCRP, mg/L | 3.4 (2.8-4.0) | 4.1 (3.4-4.7) | 1.6 (0.8-2.3) | 4.4 (3.1-5.8) |
| PREVENT 10-yr, % | 0.0 (0.0-0.2) | 54.8 (49.9-59.7) | 0.0 (0.0-0.0) | 5.2 (3.0-8.8) |
| PREVENT 30-yr, % | 92.6 (89.0-95.1) | 9.5 (6.4-13.8) | 1.5 (0.4-5.6) | 71.5 (62.7-79.0) |
| Current Smoker, % | 14.8 (12.0-18.2) | 17.6 (13.6-22.4) | 27.3 (16.1-42.4) | 20.8 (16.2-26.2) |
| Hypertension, % | 12.8 (10.3-15.8) | 45.6 (41.8-49.5) | 25.4 (14.3-41.2) | 31.1 (23.5-39.8) |
| Antihypertensives, % | 4.7 (3.6-6.1) | 24.0 (21.0-27.3) | 15.3 (7.1-29.9) | 11.5 (6.8-18.9) |
| Diabetes Mellitus, % | 0.1 (0.0-0.2) | 41.3 (36.9-45.9) | 0.0 (0.0-0.0) | 5.9 (3.0-11.3) |
| Antihyperglycemics, % | 0.0 (0.0-0.2) | 9.0 (6.7-12.0) | 0.0 (0.0-0.0) | 2.6 (0.7-8.9) |
| Prior ASCVD, % | 0.0 (0.0-0.0) | 23.9 (19.8-28.4) | 0.0 (0.0-0.0) | 0.0 (0.0-0.0) |

**Supplementary Table 4.** Logistic regression results from univariate analyses with the outcome of new eligibility under the 2026 guidelines. Results are reported as odds ratios with 95% confidence intervals.

|  | New Eligibility under 2026 Guidelines |
| --- | --- |
| Age, per decade increase | 2.31 (1.95-2.74) |
| Female Sex [Reference: Male Sex] | 0.77 (0.56-1.07) |
|  |  |
| ***Race/Ethnicity [Reference: Non-Hispanic White]*** |  |
| Mexican American | 0.66 (0.36-1.21) |
| Non-Hispanic Asian | 0.59 (0.29-1.20) |
| Non-Hispanic Black | 0.86 (0.58-1.27) |
| Other Hispanic | 0.76 (0.40-1.45) |
| Other/Multiracial | 0.74 (0.26-2.14) |
|  |  |
| ***Education Level [Reference: Less than High School]*** |  |
| Some College or AA Degree | 0.68 (0.42-1.10) |
| College Graduate or Above | 0.78 (0.50-1.21) |
|  |  |
| ***Marital Status [Reference: Married]*** |  |
| Single/Never Married | 0.54 (0.31-0.95) |
| Widowed/Divorced | 1.69 (1.01-2.83) |
|  |  |
| No Health Insurance [Reference: Insured] | 1.13 (0.64-1.99) |
| Unemployed [Reference: Employed] | 1.78 (1.14-2.80) |
| Family PIR, per SD increase | 1.01 (0.80-1.28) |
|  |  |
| ***Clinical Characteristics*** |  |
| BMI, per SD increase | 1.24 (1.06-1.45) |
| Total Cholesterol, per SD increase | 1.74 (1.31-2.32) |
| Current Smoker | 1.45 (1.04-2.02) |
| eGFR, per SD increase | 0.59 (0.49-0.71) |
| Hypertension Diagnosis | 2.89 (2.04-4.11) |
| Antihypertensive Medication | 2.38 (1.19-4.77) |

**Supplementary Table 5.** Logistic regression results from multivariable analysis with the outcome of new eligibility under the 2026 guidelines. Results are reported as odds ratios with 95% confidence intervals.

|  | New Eligibility under 2026 Guidelines |
| --- | --- |
| Age, per decade increase | 3.08 (2.43-3.90) |
| Female Sex [Reference: Male Sex] | 0.30 (0.17-0.51) |
|  |  |
| ***Race/Ethnicity [Reference: Non-Hispanic White]*** |  |
| Mexican American | 1.04 (0.60-1.79) |
| Non-Hispanic Asian | 1.06 (0.52-2.16) |
| Non-Hispanic Black | 0.81 (0.50-1.31) |
| Other Hispanic | 1.50 (0.74-3.04) |
| Other/Multiracial | 0.93 (0.27-3.18) |
|  |  |
| ***Education Level [Reference: Less than High School]*** |  |
| Some College or AA Degree | 0.74 (0.38-1.46) |
| College Graduate or Above | 1.30 (0.69-2.44) |
|  |  |
| ***Marital Status [Reference: Married]*** |  |
| Single/Never Married | 0.64 (0.31-1.33) |
| Widowed/Divorced | 1.19 (0.57-2.47) |
|  |  |
| No Health Insurance [Reference: Insured] | 1.91 (0.92-3.97) |
| Unemployed [Reference: Employed] | 1.27 (0.78-2.07) |
| Family PIR, per SD increase | 1.13 (0.88-1.45) |
|  |  |
| ***Clinical Characteristics*** |  |
| BMI, per SD increase | 1.69 (1.30-2.19) |
| Total Cholesterol, per SD increase | 1.85 (1.43-2.41) |
| Current Smoker | 4.18 (2.37-7.37) |
| eGFR, per SD increase | 0.71 (0.56-0.90) |
| Hypertension Diagnosis | 3.34 (1.65-6.74) |
| Antihypertensive Medication | 0.73 (0.22-2.44) |

| **Supplemental Table 6** | | | | |
| --- | --- | --- | --- | --- |
| **Sensitivity – LDL: NIH Equation 2 Calculation** | **Not Eligible with 2018 or 2026** | **Eligible with both 2018 and 2026** | **Eligible with 2018 Only** | **Eligible with 2026 Only** |
| Unweighted Sample Size | 1009 | 1308 | 87 | 362 |
| Weighted Sample Size, millions | 34.0 | 29.6 | 2.0 | 13.0 |
| Age, yrs | 43.1 (41.9-44.4) | 62.2 (61.2-63.2) | 58.2 (55.7-60.7) | 52.0 (50.6-53.4) |
| Women, % | 59.3 (53.4-64.9) | 41.7 (37.5-46.0) | 27.3 (14.5-45.3) | 50.3 (42.6-58.1) |
|  |  |  |  |  |
| *Sociodemographic Characteristics* |  |  |  |  |
| Race/Ethnicity |  |  |  |  |
| Mexican American, % | 9.2 (6.3-13.2) | 6.7 (5.0-8.9) | 4.8 (2.3-9.9) | 6.9 (4.7-10.1) |
| Non-Hispanic Asian, % | 6.0 (4.1-8.7) | 5.4 (3.9-7.4) | 8.9 (3.8-19.4) | 4.0 (2.1-7.7) |
| Non-Hispanic Black, % | 9.0 (6.4-12.7) | 12.0 (8.8-16.0) | 13.4 (6.9-24.4) | 8.8 (6.0-12.6) |
| Non-Hispanic White, % | 64.1 (57.2-70.4) | 63.8 (57.6-69.6) | 63.1 (42.3-80.0) | 70.5 (65.3-75.2) |
| Other Hispanic, % | 6.9 (4.8-9.6) | 7.7 (5.9-10.1) | 5.3 (1.7-15.2) | 5.7 (3.7-8.8) |
| Other/Multiracial, % | 4.9 (3.1-7.4) | 4.4 (3.0-6.4) | 4.5 (0.7-23.6) | 4.1 (1.6-9.7) |
| Education Level |  |  |  |  |
| High School Graduate or Less, % | 27.9 (23.8-32.5) | 46.3 (42.4-50.3) | 40.7 (24.3-59.5) | 35.9 (27.1-45.8) |
| Some College or AA Degree, % | 31.7 (26.7-37.1) | 26.4 (22.6-30.5) | 38.0 (25.9-51.9) | 27.7 (19.3-37.9) |
| College Graduate or Above, % | 40.4 (34.8-46.3) | 27.3 (22.3-33.0) | 21.3 (11.6-35.7) | 36.4 (27.3-46.6) |
| No Health Insurance, % | 13.2 (10.2-16.8) | 8.7 (6.1-12.2) | 13.9 (5.2-32.3) | 14.7 (9.3-22.3) |
| Currently Unemployed, % | 19.2 (16.9-21.8) | 53.9 (50.1-57.7) | 35.0 (20.7-52.6) | 30.8 (22.1-41.1) |
| Family PIR, a.u. | 3.4 (3.2-3.6) | 3.1 (2.9-3.2) | 3.1 (2.4-3.7) | 3.3 (3.0-3.6) |
|  |  |  |  |  |
| *Clinical Measures* |  |  |  |  |
| Weight, kg | 83.2 (80.5-85.9) | 88.5 (87.1-89.9) | 78.2 (74.0-82.4) | 88.2 (84.6-91.8) |
| Height, cm | 168.7 (167.7-169.7) | 168.3 (167.3-169.2) | 169.3 (165.9-172.7) | 169.9 (168.2-171.6) |
| Body Mass Index, kg/m2 | 29.1 (28.3-29.9) | 31.2 (30.6-31.8) | 27.2 (26.2-28.2) | 30.6 (29.7-31.4) |
| SBP, mmHg | 115.1 (113.9-116.4) | 130.1 (128.5-131.6) | 125.9 (121.9-129.9) | 124.8 (122.4-127.1) |
| DBP, mmHg | 73.5 (72.4-74.6) | 75.7 (74.5-76.9) | 76.7 (74.6-78.8) | 78.4 (77.0-79.9) |
| Total Cholesterol, mg/dL | 186.0 (182.4-189.6) | 190.4 (183.3-197.5) | 207.5 (197.4-217.5) | 201.9 (197.6-206.2) |
| Triglycerides, mg/dL | 93.7 (89.4-98.1) | 132.4 (121.7-143.1) | 115.2 (74.6-155.7) | 138.0 (123.4-152.5) |
| HDL, mg/dL | 57.6 (55.8-59.4) | 51.0 (49.4-52.6) | 57.1 (51.8-62.4) | 51.0 (48.6-53.3) |
| Non-HDL-c, mg/dL | 128.5 (124.6-132.3) | 139.4 (132.4-146.4) | 150.4 (140.8-160.0) | 150.9 (147.0-154.9) |
| HbA1c, % | 5.3 (5.3-5.4) | 6.3 (6.2-6.4) | 5.5 (5.5-5.6) | 5.7 (5.6-5.8) |
| eGFR, mL/min/1.73m2 | 101.1 (99.3-103.0) | 85.8 (83.5-88.1) | 95.5 (90.7-100.3) | 92.6 (90.5-94.7) |
| UACR, a.u. | 17.6 (7.1-28.2) | 46.8 (32.1-61.5) | 7.8 (4.6-10.9) | 16.0 (10.2-21.8) |
| hsCRP, mg/L | 3.4 (2.8-4.0) | 4.3 (3.7-4.9) | 1.5 (0.9-2.2) | 4.5 (3.2-5.7) |
| PREVENT 10-yr, % | 0.0 (0.0-0.2) | 49.0 (45.5-52.6) | 0.0 (0.0-0.0) | 5.3 (3.1-8.8) |
| PREVENT 30-yr, % | 91.7 (87.9-94.5) | 11.8 (9.4-14.7) | 13.6 (6.1-27.5) | 73.8 (66.1-80.3) |
| Current Smoker, % | 14.8 (11.9-18.1) | 18.8 (15.7-22.4) | 22.5 (13.4-35.2) | 20.6 (15.8-26.5) |
| Hypertension, % | 13.4 (10.6-16.7) | 45.2 (41.6-48.8) | 21.3 (11.2-36.7) | 29.3 (22.7-36.8) |
| Antihypertensives, % | 4.7 (3.6-6.1) | 22.7 (20.2-25.5) | 13.0 (6.0-26.0) | 11.4 (6.8-18.5) |
| Diabetes Mellitus, % | 0.1 (0.1-0.3) | 41.0 (37.1-45.0) | 0.0 (0.0-0.0) | 6.3 (3.4-11.6) |
| Antihyperglycemics, % | 0.0 (0.0-0.2) | 7.2 (5.4-9.6) | 0.0 (0.0-0.0) | 2.5 (0.7-8.9) |
| Prior ASCVD, % | 0.0 (0.0-0.0) | 23.9 (19.8-28.4) | 0.0 (0.0-0.0) | 0.0 (0.0-0.0) |

| **Supplemental Table 7** | | | | |
| --- | --- | --- | --- | --- |
| **Sensitivity – LDL: Friedewald Calculation** | **Not Eligible with 2018 or 2026** | **Eligible with both 2018 and 2026** | **Eligible with 2018 Only** | **Eligible with 2026 Only** |
| Unweighted Sample Size | 1012 | 1293 | 84 | 358 |
| Weighted Sample Size, millions | 34.2 | 29.1 | 1.9 | 12.6 |
| Age, yrs | 43.1 (41.9-44.4) | 62.3 (61.2-63.3) | 59.4 (57.8-61.0) | 52.3 (50.9-53.8) |
| Women, % | 59.2 (53.4-64.8) | 41.7 (37.4-46.0) | 25.8 (13.7-43.1) | 51.8 (43.9-59.6) |
|  |  |  |  |  |
| *Sociodemographic Characteristics* |  |  |  |  |
| Race/Ethnicity |  |  |  |  |
| Mexican American, % | 9.1 (6.3-13.1) | 6.6 (5.0-8.7) | 5.2 (2.5-10.2) | 7.0 (4.9-9.9) |
| Non-Hispanic Asian, % | 6.0 (4.1-8.8) | 5.5 (3.9-7.6) | 8.7 (3.7-19.3) | 4.1 (2.1-7.9) |
| Non-Hispanic Black, % | 9.0 (6.3-12.6) | 12.1 (9.0-16.2) | 14.4 (7.5-25.9) | 9.0 (6.1-13.2) |
| Non-Hispanic White, % | 64.2 (57.3-70.5) | 63.6 (57.6-69.2) | 61.2 (40.8-78.3) | 69.7 (64.5-74.6) |
| Other Hispanic, % | 6.8 (4.8-9.6) | 7.8 (6.0-10.2) | 5.7 (1.9-15.9) | 5.9 (3.9-8.9) |
| Other/Multiracial, % | 4.8 (3.1-7.4) | 4.4 (3.0-6.5) | 4.8 (0.8-25.0) | 4.2 (1.7-9.9) |
| Education Level |  |  |  |  |
| High School Graduate or Less, % | 27.8 (23.7-32.4) | 46.3 (42.3-50.3) | 43.7 (24.8-64.6) | 36.6 (28.0-46.2) |
| Some College or AA Degree, % | 31.9 (26.9-37.4) | 26.4 (22.6-30.7) | 34.3 (20.8-50.9) | 26.6 (19.1-35.8) |
| College Graduate or Above, % | 40.3 (34.7-46.1) | 27.3 (22.2-33.0) | 22.0 (12.2-36.4) | 36.8 (27.9-46.7) |
| No Health Insurance, % | 13.3 (10.3-17.0) | 8.7 (6.1-12.2) | 12.3 (3.8-33.4) | 15.1 (9.8-22.6) |
| Currently Unemployed, % | 19.3 (16.9-21.9) | 53.7 (49.8-57.7) | 34.9 (21.2-51.6) | 31.2 (22.7-41.2) |
| Family PIR, a.u. | 3.4 (3.2-3.6) | 3.1 (2.9-3.2) | 3.0 (2.4-3.6) | 3.3 (3.0-3.7) |
|  |  |  |  |  |
| *Clinical Measures* |  |  |  |  |
| Weight, kg | 83.2 (80.5-85.9) | 88.6 (87.2-90.0) | 77.5 (73.6-81.5) | 87.9 (84.2-91.6) |
| Height, cm | 168.7 (167.7-169.7) | 168.3 (167.4-169.2) | 169.0 (166.0-172.0) | 169.6 (167.8-171.4) |
| Body Mass Index, kg/m2 | 29.1 (28.3-29.9) | 31.2 (30.6-31.8) | 27.1 (26.0-28.2) | 30.6 (29.6-31.5) |
| SBP, mmHg | 115.2 (113.9-116.4) | 130.1 (128.5-131.7) | 126.4 (122.2-130.6) | 124.8 (122.5-127.2) |
| DBP, mmHg | 73.5 (72.4-74.6) | 75.7 (74.5-76.8) | 76.4 (74.2-78.7) | 78.4 (77.0-79.9) |
| Total Cholesterol, mg/dL | 186.4 (182.8-190.0) | 189.6 (182.9-196.2) | 202.6 (191.3-213.9) | 200.1 (194.4-205.9) |
| Triglycerides, mg/dL | 93.7 (89.3-98.0) | 127.0 (118.5-135.5) | 118.2 (75.3-161.2) | 123.4 (113.9-132.9) |
| HDL, mg/dL | 57.6 (55.8-59.4) | 51.2 (49.6-52.8) | 56.1 (51.1-61.1) | 51.3 (48.9-53.6) |
| Non-HDL-c, mg/dL | 128.8 (124.8-132.7) | 138.3 (132.0-144.7) | 146.5 (134.5-158.4) | 148.9 (143.4-154.3) |
| HbA1c, % | 5.3 (5.3-5.4) | 6.3 (6.1-6.4) | 5.6 (5.5-5.7) | 5.7 (5.6-5.8) |
| eGFR, mL/min/1.73m2 | 101.1 (99.3-103.0) | 85.7 (83.4-88.0) | 95.3 (90.8-99.8) | 92.9 (90.9-94.9) |
| UACR, a.u. | 17.6 (7.1-28.1) | 46.8 (32.0-61.7) | 8.1 (4.8-11.3) | 13.7 (9.4-18.1) |
| hsCRP, mg/L | 3.4 (2.8-4.0) | 4.3 (3.7-4.9) | 1.5 (0.8-2.2) | 4.5 (3.2-5.9) |
| PREVENT 10-yr, % | 0.0 (0.0-0.2) | 49.0 (45.7-52.3) | 0.0 (0.0-0.0) | 5.3 (3.1-8.9) |
| PREVENT 30-yr, % | 91.8 (87.9-94.5) | 11.9 (9.4-14.8) | 7.2 (1.7-25.3) | 73.0 (65.4-79.5) |
| Current Smoker, % | 14.7 (11.9-18.0) | 18.6 (15.4-22.4) | 24.2 (13.5-39.3) | 21.1 (16.3-26.9) |
| Hypertension, % | 13.3 (10.6-16.6) | 45.1 (41.5-48.7) | 22.9 (12.4-38.4) | 30.3 (23.2-38.4) |
| Antihypertensives, % | 4.7 (3.5-6.1) | 22.6 (20.2-25.3) | 13.9 (6.5-27.5) | 11.7 (7.0-19.1) |
| Diabetes Mellitus, % | 0.1 (0.1-0.3) | 41.2 (37.4-45.0) | 0.0 (0.0-0.0) | 6.2 (3.2-11.7) |
| Antihyperglycemics, % | 0.0 (0.0-0.2) | 7.3 (5.5-9.8) | 0.0 (0.0-0.0) | 2.6 (0.7-9.1) |
| Prior ASCVD, % | 0.0 (0.0-0.0) | 23.9 (19.8-28.4) | 0.0 (0.0-0.0) | 0.0 (0.0-0.0) |

| **Supplemental Table 8** | | | | |
| --- | --- | --- | --- | --- |
| **Sensitivity – PREVENT: with HbA1c** | **Not Eligible with 2018 or 2026** | **Eligible with both 2018 and 2026** | **Eligible with 2018 Only** | **Eligible with 2026 Only** |
| Unweighted Sample Size | 949 | 1372 | 4 | 422 |
| Weighted Sample Size, millions | 32.3 | 30.9 | 0.1 | 14.5 |
| Age, yrs | 41.3 (40.4-42.3) | 62.1 (61.0-63.2) | 46.4 (39.0-53.8) | 55.3 (53.9-56.7) |
| Women, % | 54.0 (47.8-60.1) | 40.7 (36.7-45.0) | 0.0 (0.0-0.0) | 64.5 (57.9-70.5) |
|  |  |  |  |  |
| *Sociodemographic Characteristics* |  |  |  |  |
| Race/Ethnicity |  |  |  |  |
| Mexican American, % | 9.5 (6.5-13.6) | 6.5 (4.9-8.5) | 29.3 (3.3-83.3) | 6.3 (4.3-9.3) |
| Non-Hispanic Asian, % | 6.3 (4.4-8.9) | 5.6 (4.0-7.9) | 0.0 (0.0-0.0) | 3.9 (2.0-7.2) |
| Non-Hispanic Black, % | 9.4 (6.8-12.8) | 12.1 (9.0-16.0) | 70.7 (16.7-96.7) | 8.3 (5.2-12.9) |
| Non-Hispanic White, % | 62.5 (56.4-68.2) | 63.6 (57.4-69.4) | 0.0 (0.0-0.0) | 72.7 (66.8-77.9) |
| Other Hispanic, % | 6.9 (4.8-9.8) | 7.7 (5.8-10.1) | 0.0 (0.0-0.0) | 5.9 (4.0-8.6) |
| Other/Multiracial, % | 5.4 (3.5-8.4) | 4.5 (3.0-6.7) | 0.0 (0.0-0.0) | 2.9 (1.3-6.5) |
| Education Level |  |  |  |  |
| High School Graduate or Less, % | 27.4 (23.1-32.2) | 46.2 (42.7-49.9) | 17.8 (1.8-71.6) | 36.3 (30.3-42.8) |
| Some College or AA Degree, % | 29.1 (23.9-34.8) | 26.8 (23.2-30.6) | 73.1 (24.4-95.8) | 33.6 (26.9-41.0) |
| College Graduate or Above, % | 43.5 (37.2-50.2) | 27.0 (22.2-32.4) | 9.1 (0.9-52.9) | 30.1 (22.7-38.6) |
| No Health Insurance, % | 14.7 (11.9-18.1) | 8.9 (6.4-12.3) | 0.0 (0.0-0.0) | 11.7 (7.0-19.0) |
| Currently Unemployed, % | 16.4 (13.9-19.3) | 52.6 (48.1-57.1) | 17.8 (1.8-71.6) | 36.2 (29.5-43.6) |
| Family PIR, a.u. | 3.4 (3.2-3.5) | 3.1 (2.9-3.2) | 3.1 (2.4-3.8) | 3.4 (3.1-3.8) |
|  |  |  |  |  |
| *Clinical Measures* |  |  |  |  |
| Weight, kg | 83.8 (81.1-86.5) | 87.9 (86.5-89.3) | 106.1 (94.3-117.9) | 85.9 (82.4-89.3) |
| Height, cm | 169.3 (168.2-170.4) | 168.3 (167.5-169.2) | 175.4 (170.3-180.4) | 168.2 (166.8-169.7) |
| Body Mass Index, kg/m2 | 29.1 (28.3-29.9) | 31.0 (30.4-31.6) | 34.6 (29.2-40.0) | 30.2 (29.2-31.2) |
| SBP, mmHg | 115.0 (113.7-116.4) | 129.8 (128.3-131.4) | 143.3 (128.5-158.1) | 123.8 (121.5-126.1) |
| DBP, mmHg | 73.7 (72.8-74.7) | 75.7 (74.6-76.8) | 99.6 (87.8-111.4) | 77.4 (75.3-79.4) |
| Total Cholesterol, mg/dL | 184.8 (181.5-188.1) | 190.3 (184.0-196.6) | 178.2 (155.2-201.1) | 202.0 (195.0-209.0) |
| Triglycerides, mg/dL | 94.2 (88.9-99.5) | 126.6 (118.1-135.1) | 70.9 (69.1-72.8) | 118.1 (111.7-124.4) |
| HDL, mg/dL | 56.5 (54.5-58.5) | 51.4 (50.0-52.9) | 66.8 (60.6-73.0) | 54.7 (52.4-57.0) |
| Non-HDL-c, mg/dL | 128.3 (124.5-132.2) | 138.8 (132.7-145.0) | 111.4 (82.3-140.5) | 147.3 (141.8-152.7) |
| HbA1c, % | 5.3 (5.3-5.3) | 6.3 (6.1-6.4) | 5.4 (5.1-5.6) | 5.7 (5.6-5.8) |
| eGFR, mL/min/1.73m2 | 101.8 (99.7-103.9) | 86.3 (84.0-88.5) | 100.8 (87.1-114.5) | 92.3 (90.3-94.3) |
| UACR, a.u. | 17.8 (6.9-28.8) | 44.6 (30.6-58.5) | 4.7 (2.3-7.2) | 13.6 (9.5-17.6) |
| hsCRP, mg/L | 3.2 (2.5-4.0) | 4.1 (3.6-4.7) | 1.5 (1.4-1.7) | 4.7 (3.3-6.2) |
| PREVENT 10-yr, % | 1.9 (1.7-2.0) | 13.3 (12.6-14.0) | 3.9 (3.8-4.0) | 6.3 (6.0-6.7) |
| PREVENT 30-yr, % | 8.6 (8.1-9.1) | 27.0 (26.2-27.8) | 15.6 (14.2-17.1) | 20.9 (20.3-21.5) |
| Current Smoker, % | 15.5 (12.8-18.6) | 19.0 (15.7-22.7) | 9.1 (0.9-52.9) | 18.6 (14.6-23.5) |
| Hypertension, % | 12.3 (9.4-15.9) | 43.7 (40.1-47.3) | 100.0 (100.0-100.0) | 30.4 (22.1-40.1) |
| Antihypertensives, % | 4.7 (3.5-6.4) | 22.0 (19.7-24.5) | 82.2 (28.4-98.2) | 10.7 (6.6-16.9) |
| Diabetes Mellitus, % | 0.2 (0.1-0.3) | 38.8 (35.1-42.6) | 0.0 (0.0-0.0) | 5.3 (2.7-10.2) |
| Antihyperglycemics, % | 0.0 (0.0-0.2) | 6.9 (5.1-9.2) | 0.0 (0.0-0.0) | 2.3 (0.6-8.0) |
| Prior ASCVD, % | 0.0 (0.0-0.0) | 23.9 (19.8-28.4) | 0.0 (0.0-0.0) | 0.0 (0.0-0.0) |

| **Supplemental Table 9** | | | | |
| --- | --- | --- | --- | --- |
| **Sensitivity – PREVENT: with UACR** | **Not Eligible with 2018 or 2026** | **Eligible with both 2018 and 2026** | **Eligible with 2018 Only** | **Eligible with 2026 Only** |
| Unweighted Sample Size | 1009 | 1284 | 92 | 362 |
| Weighted Sample Size, millions | 33.3 | 29.1 | 1.9 | 13.4 |
| Age, yrs | 45.2 (44.0-46.5) | 62.9 (61.2-64.6) | 66.2 (65.2-67.3) | 56.1 (54.6-57.5) |
| Women, % | 60.8 (54.8-66.4) | 41.8 (37.5-46.2) | 23.3 (12.2-39.8) | 48.5 (42.2-54.9) |
|  |  |  |  |  |
| *Sociodemographic Characteristics* |  |  |  |  |
| Race/Ethnicity |  |  |  |  |
| Mexican American, % | 9.4 (6.4-13.5) | 6.4 (4.8-8.6) | 8.1 (4.8-13.3) | 6.4 (4.4-9.2) |
| Non-Hispanic Asian, % | 6.2 (4.2-9.0) | 5.6 (4.0-7.7) | 6.5 (2.5-16.2) | 4.0 (2.1-7.4) |
| Non-Hispanic Black, % | 9.3 (6.6-13.0) | 12.1 (9.0-16.0) | 14.6 (9.1-22.6) | 8.3 (5.5-12.3) |
| Non-Hispanic White, % | 63.1 (56.1-69.6) | 63.7 (57.7-69.4) | 59.2 (41.9-74.5) | 72.0 (66.1-77.3) |
| Other Hispanic, % | 7.0 (4.9-10.0) | 7.7 (5.9-10.1) | 6.8 (2.6-16.4) | 5.4 (3.3-8.7) |
| Other/Multiracial, % | 5.0 (3.2-7.6) | 4.5 (3.0-6.5) | 4.7 (0.8-23.7) | 3.9 (1.6-9.4) |
| Education Level |  |  |  |  |
| High School Graduate or Less, % | 28.1 (24.5-32.0) | 46.8 (42.5-51.1) | 37.0 (22.6-54.2) | 35.2 (27.2-44.1) |
| Some College or AA Degree, % | 31.5 (26.8-36.8) | 26.4 (22.6-30.6) | 33.6 (19.0-52.3) | 27.9 (20.6-36.6) |
| College Graduate or Above, % | 40.4 (35.1-45.9) | 26.8 (21.6-32.7) | 29.4 (18.7-43.0) | 36.9 (27.4-47.5) |
| No Health Insurance, % | 13.5 (10.5-17.3) | 8.5 (5.9-12.0) | 15.0 (5.7-33.8) | 14.3 (8.6-22.9) |
| Currently Unemployed, % | 19.7 (17.2-22.6) | 53.6 (49.4-57.7) | 36.8 (23.6-52.4) | 29.5 (21.3-39.2) |
| Family PIR, a.u. | 3.3 (3.1-3.5) | 2.9 (2.7-3.1) | 2.1 (1.1-3.0) | 3.2 (2.7-3.7) |
|  |  |  |  |  |
| *Clinical Measures* |  |  |  |  |
| Weight, kg | 77.0 (74.0-79.9) | 83.8 (80.5-87.2) | 69.2 (61.4-77.0) | 82.2 (77.8-86.7) |
| Height, cm | 162.8 (162.0-163.7) | 159.7 (158.8-160.5) | 157.6 (154.5-160.6) | 163.2 (162.1-164.2) |
| Body Mass Index, kg/m2 | 28.9 (27.8-30.1) | 32.8 (31.5-34.1) | 27.7 (25.4-29.9) | 31.0 (29.3-32.6) |
| SBP, mmHg | 112.2 (110.7-113.7) | 131.2 (128.6-133.7) | 129.9 (123.1-136.8) | 125.6 (121.9-129.2) |
| DBP, mmHg | 72.2 (70.9-73.5) | 75.2 (73.6-76.8) | 76.1 (70.3-82.0) | 78.0 (75.3-80.6) |
| Total Cholesterol, mg/dL | 188.5 (184.3-192.7) | 197.3 (187.7-206.9) | 221.1 (204.5-237.7) | 201.7 (191.9-211.4) |
| Triglycerides, mg/dL | 87.1 (81.9-92.3) | 124.9 (114.7-135.2) | 89.0 (72.6-105.4) | 111.1 (99.8-122.4) |
| HDL, mg/dL | 62.4 (60.4-64.4) | 56.1 (53.4-58.8) | 72.3 (63.3-81.3) | 54.7 (52.4-56.9) |
| Non-HDL-c, mg/dL | 126.1 (121.9-130.4) | 141.3 (131.3-151.2) | 148.8 (130.1-167.6) | 147.0 (137.3-156.7) |
| HbA1c, % | 5.4 (5.3-5.4) | 6.3 (6.2-6.5) | 5.6 (5.5-5.8) | 5.7 (5.6-5.9) |
| eGFR, mL/min/1.73m2 | 99.9 (97.7-102.1) | 84.9 (81.3-88.6) | 81.3 (74.1-88.5) | 90.9 (88.6-93.3) |
| UACR, a.u. | 15.4 (7.9-22.9) | 59.2 (27.4-91.0) | 7.3 (4.9-9.6) | 14.9 (10.1-19.8) |
| hsCRP, mg/L | 3.7 (2.9-4.6) | 5.4 (4.5-6.2) | 1.4 (1.1-1.8) | 5.5 (3.8-7.1) |
| PREVENT 10-yr, % | 1.0 (0.9-1.1) | 7.2 (6.2-8.3) | 4.1 (3.9-4.3) | 2.9 (2.7-3.2) |
| PREVENT 30-yr, % | 4.8 (4.3-5.2) | 16.1 (14.9-17.2) | 13.4 (12.8-13.9) | 11.4 (11.0-11.9) |
| Current Smoker, % | 15.1 (12.2-18.5) | 19.1 (15.8-22.9) | 16.1 (9.5-26.2) | 19.8 (15.3-25.4) |
| Hypertension, % | 11.8 (9.6-14.5) | 44.1 (40.6-47.7) | 38.5 (25.8-53.1) | 32.8 (23.7-43.5) |
| Antihypertensives, % | 4.5 (3.6-5.7) | 21.6 (19.2-24.1) | 30.1 (17.9-46.0) | 11.7 (7.3-18.1) |
| Diabetes Mellitus, % | 0.2 (0.1-0.3) | 41.2 (37.2-45.4) | 0.0 (0.0-0.0) | 5.7 (2.9-10.9) |
| Antihyperglycemics, % | 0.0 (0.0-0.2) | 7.3 (5.4-9.8) | 0.0 (0.0-0.0) | 2.5 (0.7-8.6) |
| Prior ASCVD, % | 0.0 (0.0-0.0) | 23.9 (19.8-28.4) | 0.0 (0.0-0.0) | 0.0 (0.0-0.0) |

| **Supplemental Table 10** | | | | |
| --- | --- | --- | --- | --- |
| **Sensitivity – PREVENT: with HbA1c, UACR, and SDI** | **Not Eligible with 2018 or 2026** | **Eligible with both 2018 and 2026** | **Eligible with 2018 Only** | **Eligible with 2026 Only** |
| Unweighted Sample Size | 1023 | 1353 | 23 | 348 |
| Weighted Sample Size, millions | 34.2 | 30.5 | 0.5 | 12.6 |
| Age, yrs | 42.5 (41.5-43.5) | 62.2 (61.1-63.3) | 52.7 (51.2-54.2) | 54.3 (52.5-56.1) |
| Women, % | 56.9 (51.0-62.5) | 41.3 (37.1-45.6) | 1.5 (0.1-17.3) | 58.2 (52.1-64.0) |
|  |  |  |  |  |
| *Sociodemographic Characteristics* |  |  |  |  |
| Race/Ethnicity |  |  |  |  |
| Mexican American, % | 9.6 (6.7-13.7) | 6.6 (5.0-8.6) | 3.6 (0.4-24.1) | 5.5 (3.5-8.5) |
| Non-Hispanic Asian, % | 6.2 (4.3-8.8) | 5.5 (3.9-7.6) | 16.3 (5.4-40.0) | 3.7 (2.1-6.7) |
| Non-Hispanic Black, % | 9.5 (6.9-12.8) | 12.1 (9.0-16.1) | 20.7 (8.0-44.0) | 7.8 (4.5-13.2) |
| Non-Hispanic White, % | 62.2 (56.3-67.7) | 64.0 (57.8-69.7) | 30.2 (5.5-76.1) | 75.1 (69.4-80.0) |
| Other Hispanic, % | 7.2 (5.3-9.8) | 7.5 (5.6-9.9) | 19.3 (6.7-44.2) | 4.8 (3.1-7.4) |
| Other/Multiracial, % | 5.3 (3.5-7.8) | 4.4 (2.9-6.6) | 10.0 (1.6-42.3) | 3.0 (1.4-6.3) |
| Education Level |  |  |  |  |
| High School Graduate or Less, % | 28.0 (23.7-32.8) | 46.1 (42.6-49.6) | 55.2 (25.3-81.8) | 35.8 (27.7-44.8) |
| Some College or AA Degree, % | 30.8 (25.7-36.5) | 26.9 (23.3-31.0) | 22.0 (7.2-50.5) | 29.6 (22.6-37.7) |
| College Graduate or Above, % | 41.1 (35.2-47.3) | 27.0 (22.1-32.5) | 22.7 (6.7-54.6) | 34.6 (25.9-44.4) |
| No Health Insurance, % | 13.8 (10.8-17.5) | 9.0 (6.5-12.4) | 0.0 (0.0-0.0) | 13.7 (8.4-21.6) |
| Currently Unemployed, % | 18.7 (15.8-22.0) | 53.2 (48.6-57.7) | 10.5 (3.1-29.8) | 32.9 (26.7-39.8) |
| Family PIR, a.u. | 3.4 (3.2-3.5) | 3.1 (2.9-3.2) | 2.9 (2.1-3.6) | 3.4 (3.0-3.8) |
|  |  |  |  |  |
| *Clinical Measures* |  |  |  |  |
| Weight, kg | 83.8 (81.3-86.4) | 88.1 (86.6-89.5) | 78.7 (68.5-88.9) | 86.2 (82.6-89.8) |
| Height, cm | 168.9 (167.9-169.9) | 168.3 (167.4-169.2) | 171.1 (166.2-176.1) | 169.1 (167.6-170.5) |
| Body Mass Index, kg/m2 | 29.2 (28.5-30.0) | 31.0 (30.4-31.7) | 27.1 (22.7-31.4) | 30.1 (29.1-31.2) |
| SBP, mmHg | 115.6 (114.3-116.9) | 129.9 (128.3-131.4) | 128.9 (123.3-134.5) | 123.8 (121.4-126.1) |
| DBP, mmHg | 73.9 (72.8-75.1) | 75.7 (74.6-76.8) | 78.7 (76.2-81.2) | 77.4 (75.3-79.6) |
| Total Cholesterol, mg/dL | 186.2 (183.2-189.2) | 190.3 (183.9-196.8) | 186.0 (173.1-198.9) | 201.0 (193.0-209.1) |
| Triglycerides, mg/dL | 94.1 (89.7-98.4) | 127.2 (118.6-135.8) | 87.8 (60.0-115.6) | 122.4 (115.2-129.5) |
| HDL, mg/dL | 57.0 (55.1-59.0) | 51.4 (49.9-52.9) | 55.9 (48.0-63.8) | 52.9 (50.6-55.3) |
| Non-HDL-c, mg/dL | 129.1 (125.7-132.6) | 138.9 (132.7-145.2) | 130.1 (114.0-146.2) | 148.1 (141.4-154.8) |
| HbA1c, % | 5.3 (5.3-5.4) | 6.3 (6.1-6.4) | 5.5 (5.4-5.7) | 5.7 (5.6-5.8) |
| eGFR, mL/min/1.73m2 | 101.3 (99.3-103.3) | 86.1 (83.8-88.3) | 101.3 (94.6-108.0) | 92.2 (90.1-94.3) |
| UACR, a.u. | 17.4 (7.0-27.9) | 45.1 (31.0-59.3) | 5.9 (3.7-8.2) | 14.0 (9.7-18.4) |
| hsCRP, mg/L | 3.2 (2.7-3.7) | 4.2 (3.6-4.7) | 3.6 (-1.0-8.1) | 5.1 (3.6-6.6) |
| PREVENT 10-yr, % | 0.0 (0.0-0.1) | 37.9 (33.1-42.9) | 0.0 (0.0-0.0) | 39.7 (33.3-46.5) |
| PREVENT 30-yr, % | 83.3 (78.5-87.1) | 5.1 (3.4-7.7) | 0.0 (0.0-0.0) | 27.4 (19.8-36.6) |
| Current Smoker, % | 15.5 (12.7-18.7) | 18.2 (15.1-21.9) | 67.4 (35.1-88.7) | 19.0 (15.0-23.8) |
| Hypertension, % | 13.7 (10.5-17.7) | 44.0 (40.4-47.7) | 29.8 (10.6-60.3) | 29.2 (21.5-38.2) |
| Antihypertensives, % | 5.4 (4.0-7.2) | 22.1 (19.7-24.6) | 23.5 (7.4-54.2) | 9.8 (5.6-16.5) |
| Diabetes Mellitus, % | 0.1 (0.1-0.3) | 39.3 (35.6-43.1) | 0.0 (0.0-0.0) | 6.1 (3.1-11.6) |
| Antihyperglycemics, % | 0.0 (0.0-0.2) | 7.0 (5.2-9.4) | 0.0 (0.0-0.0) | 2.6 (0.7-9.1) |
| Prior ASCVD, % | 0.0 (0.0-0.0) | 23.9 (19.8-28.4) | 0.0 (0.0-0.0) | 0.0 (0.0-0.0) |

| **Supplemental Table 11** | | | | |
| --- | --- | --- | --- | --- |
| **Sensitivity – Removed Statin Users** | **Not Eligible with 2018 or 2026** | **Eligible with both 2018 and 2026** | **Eligible with 2018 Only** | **Eligible with 2026 Only** |
| Unweighted Sample Size | 980 | 1117 | 71 | 341 |
| Weighted Sample Size, millions | 33.0 | 25.4 | 1.5 | 12.0 |
| Age, yrs | 42.7 (41.5-43.9) | 61.9 (60.8-63.1) | 59.6 (57.9-61.3) | 51.9 (50.5-53.3) |
| Women, % | 59.3 (53.1-65.1) | 41.4 (36.5-46.5) | 22.4 (10.5-41.7) | 50.3 (42.3-58.3) |
|  |  |  |  |  |
| *Sociodemographic Characteristics* |  |  |  |  |
| Race/Ethnicity |  |  |  |  |
| Mexican American, % | 9.4 (6.5-13.5) | 6.5 (4.9-8.6) | 5.3 (2.2-12.1) | 6.9 (4.7-10.0) |
| Non-Hispanic Asian, % | 6.1 (4.1-8.9) | 5.1 (3.6-7.1) | 8.9 (3.3-22.0) | 4.2 (2.1-8.2) |
| Non-Hispanic Black, % | 9.1 (6.4-12.8) | 12.2 (8.9-16.5) | 15.9 (8.2-28.5) | 9.5 (6.3-13.9) |
| Non-Hispanic White, % | 63.6 (56.8-70.0) | 63.0 (56.6-69.0) | 57.8 (35.7-77.2) | 69.0 (62.9-74.4) |
| Other Hispanic, % | 6.9 (4.8-9.7) | 8.3 (6.1-11.1) | 6.4 (2.2-16.9) | 6.1 (3.9-9.2) |
| Other/Multiracial, % | 4.9 (3.1-7.5) | 4.9 (3.2-7.3) | 5.8 (0.9-29.0) | 4.4 (1.7-10.6) |
| Education Level |  |  |  |  |
| High School Graduate or Less, % | 27.9 (23.5-32.7) | 46.8 (42.6-51.0) | 44.4 (23.4-67.5) | 36.5 (27.6-46.4) |
| Some College or AA Degree, % | 32.2 (27.0-37.9) | 25.9 (22.3-29.8) | 32.2 (16.6-53.2) | 27.3 (19.6-36.5) |
| College Graduate or Above, % | 39.9 (34.1-45.9) | 27.4 (22.5-32.9) | 23.4 (11.3-42.3) | 36.3 (27.1-46.5) |
| No Health Insurance, % | 13.7 (10.6-17.6) | 9.4 (6.5-13.4) | 13.6 (3.9-37.7) | 15.2 (9.8-22.9) |
| Currently Unemployed, % | 19.4 (16.8-22.3) | 52.6 (47.7-57.4) | 36.4 (20.8-55.5) | 31.7 (23.0-41.8) |
| Family PIR, a.u. | 3.4 (3.2-3.5) | 3.1 (2.9-3.3) | 2.8 (2.2-3.5) | 3.3 (3.0-3.7) |
|  |  |  |  |  |
| *Clinical Measures* |  |  |  |  |
| Weight, kg | 83.4 (80.7-86.1) | 88.8 (87.3-90.3) | 77.1 (72.9-81.2) | 87.8 (83.8-91.8) |
| Height, cm | 168.8 (167.7-169.8) | 168.4 (167.3-169.5) | 169.3 (165.8-172.7) | 169.9 (168.0-171.8) |
| Body Mass Index, kg/m2 | 29.1 (28.4-29.9) | 31.3 (30.6-32.0) | 26.9 (25.6-28.2) | 30.4 (29.4-31.4) |
| SBP, mmHg | 115.1 (113.7-116.5) | 130.4 (128.6-132.2) | 126.3 (122.1-130.6) | 125.0 (122.5-127.4) |
| DBP, mmHg | 73.6 (72.4-74.7) | 76.0 (74.7-77.4) | 76.1 (73.6-78.5) | 78.8 (77.4-80.2) |
| Total Cholesterol, mg/dL | 186.9 (183.3-190.5) | 193.4 (186.6-200.2) | 201.2 (190.0-212.5) | 201.5 (195.9-207.1) |
| Triglycerides, mg/dL | 94.2 (89.6-98.8) | 127.9 (118.6-137.3) | 110.3 (60.0-160.5) | 124.3 (114.4-134.2) |
| HDL, mg/dL | 57.5 (55.6-59.4) | 50.9 (49.5-52.2) | 56.1 (50.3-61.9) | 51.1 (48.6-53.5) |
| Non-HDL-c, mg/dL | 129.4 (125.4-133.4) | 142.6 (135.8-149.4) | 145.1 (132.9-157.3) | 150.4 (145.0-155.8) |
| HbA1c, % | 5.3 (5.3-5.4) | 6.2 (6.1-6.4) | 5.6 (5.5-5.7) | 5.7 (5.6-5.8) |
| eGFR, mL/min/1.73m2 | 101.4 (99.8-103.0) | 85.9 (83.4-88.4) | 94.6 (89.3-99.8) | 93.0 (90.9-95.2) |
| UACR, a.u. | 17.9 (7.1-28.7) | 42.7 (30.3-55.1) | 8.7 (4.7-12.7) | 14.1 (9.5-18.7) |
| hsCRP, mg/L | 3.5 (2.8-4.1) | 4.5 (3.8-5.2) | 1.6 (0.8-2.5) | 4.6 (3.2-6.0) |
| PREVENT 10-yr, % | 0.0 (0.0-0.2) | 50.7 (46.3-55.1) | 0.0 (0.0-0.0) | 5.4 (3.1-9.1) |
| PREVENT 30-yr, % | 92.4 (88.7-94.9) | 11.4 (8.7-14.9) | 1.1 (0.2-5.5) | 74.0 (65.9-80.8) |
| Current Smoker, % | 15.2 (12.3-18.7) | 20.1 (16.7-24.0) | 27.9 (16.0-44.1) | 21.5 (16.2-28.0) |
| Hypertension, % | 13.2 (10.7-16.2) | 47.8 (43.8-51.8) | 25.6 (14.0-42.1) | 31.0 (23.7-39.3) |
| Antihypertensives, % | 4.8 (3.7-6.3) | 25.9 (23.1-28.9) | 16.3 (7.4-32.3) | 12.4 (7.2-20.5) |
| Diabetes Mellitus, % | 0.2 (0.1-0.3) | 38.6 (35.2-42.0) | 0.0 (0.0-0.0) | 6.4 (3.2-12.1) |
| Antihyperglycemics, % | 0.0 (0.0-0.2) | 8.3 (6.2-11.1) | 0.0 (0.0-0.0) | 2.7 (0.7-9.6) |
| Prior ASCVD, % | 0.0 (0.0-0.0) | 23.9 (19.8-28.4) | 0.0 (0.0-0.0) | 0.0 (0.0-0.0) |

**Supplemental Document References**

1.	Joynt Maddox KE, Elkind MSV, Aparicio HJ, Commodore-Mensah Y, de Ferranti SD, Dowd WN, Hernandez AF, Khavjou O, Michos ED, Palaniappan L, et al. Forecasting the Burden of Cardiovascular Disease and Stroke in the United States Through 2050—Prevalence of Risk Factors and Disease: A Presidential Advisory From the American Heart Association. *Circulation*. 2024;150:e65-e88. doi: 10.1161/CIR.0000000000001256

2.	Martin SS, Aday AW, Allen NB, Almarzooq ZI, Anderson CAM, Arora P, Avery CL, Baker-Smith CM, Bansal N, Beaton AZ, et al. 2025 Heart Disease and Stroke Statistics: A Report of US and Global Data From the American Heart Association. *Circulation*.0. doi: doi:10.1161/CIR.0000000000001303

3.	Harrison DG, Touyz RM. Hypertension Editors’ View of the 2025 Guideline for the Prevention, Detection, Evaluation, and Management of High Blood Pressure in Adults. *Hypertension*.0. doi: 10.1161/HYPERTENSIONAHA.125.25467

4.	Metlock FE, Hinneh T, Benjasirisan C, Alharthi A, Ogungbe O, Turkson-Ocran RN, Himmelfarb CR, Commodore-Mensah Y. Impact of Social Determinants of Health on Hypertension Outcomes: A Systematic Review. *Hypertension*. 2024;81:1675-1700. doi: 10.1161/hypertensionaha.123.22571

5.	Whelton PK, Carey RM, Aronow WS, Casey DE, Collins KJ, Dennison Himmelfarb C, DePalma SM, Gidding S, Jamerson KA, Jones DW, et al. 2017 ACC/AHA/AAPA/ABC/ACPM/AGS/APhA/ASH/ASPC/NMA/PCNA Guideline for the Prevention, Detection, Evaluation, and Management of High Blood Pressure in Adults: A Report of the American College of Cardiology/American Heart Association Task Force on Clinical Practice Guidelines. *Hypertension*. 2018;71:e13-e115. doi: 10.1161/HYP.0000000000000065

6.	Murphy BS, Hershey MS, Huang S, Nam Y, Post WS, McClelland RL, DeFilippis AP. PREVENT Risk Score vs the Pooled Cohort Equations in MESA. *JACC: Advances*. 2025;4:101825. doi: doi:10.1016/j.jacadv.2025.101825

7.	Razavi AC, Kohli P, McGuire DK, Martin SS, Polonsky TS, McEvoy JW, Whelton SP, Blumenthal RS. PREVENT Equations: A New Era in Cardiovascular Disease Risk Assessment. *Circulation: Cardiovascular Quality and Outcomes*. 2024;17:e010763. doi: doi:10.1161/CIRCOUTCOMES.123.010763

8.	Khan SS, Matsushita K, Sang Y, Ballew SH, Grams ME, Surapaneni A, Blaha MJ, Carson AP, Chang AR, Ciemins E, et al. Development and Validation of the American Heart Association’s PREVENT Equations. *Circulation*. 2024;149:430-449. doi: doi:10.1161/CIRCULATIONAHA.123.067626

9.	Scheuermann B, Brown A, Colburn T, Hakeem H, Chow CH, Ade C. External Validation of the American Heart Association PREVENT Cardiovascular Disease Risk Equations. *JAMA Network Open*. 2024;7:e2438311-e2438311. doi: 10.1001/jamanetworkopen.2024.38311

10.	Shetty NS, Gaonkar M, Patel N, Vekariya N, Li P, Arora G, Arora P. PREVENT and Pooled Cohort Equations in Mortality Risk Prediction: National Health and Nutrition Examination Survey. *JACC Adv*. 2024;3:101372. doi: 10.1016/j.jacadv.2024.101372

11.	Jones DW, Ferdinand KC, Taler SJ, Johnson HM, Shimbo D, Abdalla M, Altieri MM, Bansal N, Bello NA, Bress AP, et al. 2025 AHA/ACC/AANP/AAPA/ABC/ACCP/ACPM/AGS/AMA/ASPC/NMA/PCNA/SGIM Guideline for the Prevention, Detection, Evaluation and Management of High Blood Pressure in Adults: A Report of the American College of Cardiology/American Heart Association Joint Committee on Clinical Practice Guidelines. *Circulation*.0. doi: 10.1161/CIR.0000000000001356

12.	Flack JM, Adekola B. Blood pressure and the new ACC/AHA hypertension guidelines. *Trends in Cardiovascular Medicine*. 2020;30:160-164. doi: [**https://doi.org/10.1016/j.tcm.2019.05.003**](https://doi.org/10.1016/j.tcm.2019.05.003)

13.	Khera R, Lu Y, Lu J, Saxena A, Nasir K, Jiang L, Krumholz HM. Impact of 2017 ACC/AHA guidelines on prevalence of hypertension and eligibility for antihypertensive treatment in United States and China: nationally representative cross sectional study. *BMJ*. 2018;362:k2357. doi: 10.1136/bmj.k2357

14.	Goff DC, Jr., Lloyd-Jones DM, Bennett G, Coady S, D'Agostino RB, Sr., Gibbons R, Greenland P, Lackland DT, Levy D, O'Donnell CJ, et al. 2013 ACC/AHA guideline on the assessment of cardiovascular risk: a report of the American College of Cardiology/American Heart Association Task Force on Practice Guidelines. *J Am Coll Cardiol*. 2014;63:2935-2959. doi: 10.1016/j.jacc.2013.11.005

15.	Benetos A, Laurent S, Hoeks AP, Boutouyrie PH, Safar ME. Arterial alterations with aging and high blood pressure. A noninvasive study of carotid and femoral arteries. *Arterioscler Thromb*. 1993;13:90-97. doi: 10.1161/01.atv.13.1.90

16.	Levey AS, Coresh J, Greene T, Marsh J, Stevens LA, Kusek JW, Van Lente F. Expressing the Modification of Diet in Renal Disease Study equation for estimating glomerular filtration rate with standardized serum creatinine values. *Clin Chem*. 2007;53:766-772. doi: 10.1373/clinchem.2006.077180

17.	Diagnosis and classification of diabetes mellitus. *Diabetes Care*. 2010;33 Suppl 1:S62-69. doi: 10.2337/dc10-S062

18.	Lees JS, Welsh CE, Celis-Morales CA, Mackay D, Lewsey J, Gray SR, Lyall DM, Cleland JG, Gill JMR, Jhund PS, et al. Glomerular filtration rate by differing measures, albuminuria and prediction of cardiovascular disease, mortality and end-stage kidney disease. *Nat Med*. 2019;25:1753-1760. doi: 10.1038/s41591-019-0627-8

19.	Diao JA, Shi I, Murthy VL, Buckley TA, Patel CJ, Pierson E, Yeh RW, Kazi DS, Wadhera RK, Manrai AK. Projected Changes in Statin and Antihypertensive Therapy Eligibility With the AHA PREVENT Cardiovascular Risk Equations. *JAMA*. 2024;332:989-1000. doi: 10.1001/jama.2024.12537

20.	Muntner P, Jaeger BC, Foti K, Ghazi L, Bundy JD, Chen L, Safford MM. Predicted Cardiovascular Risk by the PREVENT Equations in US Adults With Stage 1 Hypertension. *Hypertension*. 2024;81:1976-1985. doi: doi:10.1161/HYPERTENSIONAHA.124.22998

21.	Gulati M, Moore MM, Cibotti-Sun M. 2025 High Blood Pressure Guideline-at-a-Glance. *JACC*.0. doi: doi:10.1016/j.jacc.2025.07.010

22.	Rayani A, Sharma G, Spitz JA. Atherosclerotic Cardiovascular Disease Risk Estimates Using the New Predicting Risk of Cardiovascular Disease Events Equations: Implications for Statin Use. *Curr Cardiol Rep*. 2025;27:107. doi: 10.1007/s11886-025-02244-5

23.	Khan SS, Lloyd-Jones DM, Abdalla M, Bello NA, Blyler CA, Carter J, Commodore-Mensah Y, Ferdinand KC, Johnson HM, Jones D, et al. Use of Risk Assessment to Guide Decision-Making for Blood Pressure Management in the Primary Prevention of Cardiovascular Disease: A Scientific Statement From the American Heart Association and American College of Cardiology. *JACC*.0. doi: doi:10.1016/j.jacc.2025.08.001

24.	Jia G, Sowers JR. Hypertension in Diabetes: An Update of Basic Mechanisms and Clinical Disease. *Hypertension*. 2021;78:1197-1205. doi: 10.1161/HYPERTENSIONAHA.121.17981

25.	Sowers JR, Epstein M, Frohlich ED. Diabetes, Hypertension, and Cardiovascular Disease. *Hypertension*. 2001;37:1053-1059. doi: 10.1161/01.HYP.37.4.1053

26.	Burnier M, Damianaki A. Hypertension as Cardiovascular Risk Factor in Chronic Kidney Disease. *Circulation Research*. 2023;132:1050-1063. doi: 10.1161/CIRCRESAHA.122.321762

27.	Ndumele CE, Ferdinand KC, Khan SS. The Role of Race in Cardiovascular Disease Risk Prediction. *Circulation*. 2025;151:741-743. doi: 10.1161/circulationaha.124.071233

28.	Yan X, Bacong AM, Huang Q, Husby H, Dudum R, Jose P, Palaniappan L, Rodriguez F. Performance of the American Heart Association’s PREVENT Equations Among Disaggregated Racial and Ethnic Subgroups. *JAMA Cardiology*. 2025;10:876-885. doi: 10.1001/jamacardio.2025.1865

29.	Barzilay JI, Farag YMK, Durthaler J. Albuminuria: An Underappreciated Risk Factor for Cardiovascular Disease. *Journal of the American Heart Association*. 2024;13:e030131. doi: doi:10.1161/JAHA.123.030131

30.	Cao JJ, Barzilay JI, Peterson D, Manolio TA, Psaty BM, Kuller L, Wexler J, Bleyer AJ, Cushman M. The association of microalbuminuria with clinical cardiovascular disease and subclinical atherosclerosis in the elderly: the Cardiovascular Health Study. *Atherosclerosis*. 2006;187:372-377. doi: 10.1016/j.atherosclerosis.2005.09.015

31.	McEvoy JW, McCarthy CP, Bruno RM, Brouwers S, Canavan MD, Ceconi C, Christodorescu RM, Daskalopoulou SS, Ferro CJ, Gerdts E, et al. 2024 ESC Guidelines for the management of elevated blood pressure and hypertension: Developed by the task force on the management of elevated blood pressure and hypertension of the European Society of Cardiology (ESC) and endorsed by the European Society of Endocrinology (ESE) and the European Stroke Organisation (ESO). *European Heart Journal*. 2024;45:3912-4018. doi: 10.1093/eurheartj/ehae178

32.	McCarthy CP, Bruno RM, Rahimi K, Touyz RM, McEvoy JW. What Is New and Different in the 2024 European Society of Cardiology Guidelines for the Management of Elevated Blood Pressure and Hypertension? *Hypertension*. 2025;82:432-444. doi: doi:10.1161/HYPERTENSIONAHA.124.24173

33.	Mancia G, Kreutz R, Brunström M, Burnier M, Grassi G, Januszewicz A, Muiesan ML, Tsioufis K, Agabiti-Rosei E, Algharably EAE, et al. 2023 ESH Guidelines for the management of arterial hypertension The Task Force for the management of arterial hypertension of the European Society of Hypertension: Endorsed by the International Society of Hypertension (ISH) and the European Renal Association (ERA). *J Hypertens*. 2023;41:1874-2071. doi: 10.1097/hjh.0000000000003480

34.	Azizi M, Juraschek SP. A Trans-Atlantic Perspective on the 2025 AHA/ACC Guidelines for the Prevention, Detection, Evaluation, and Management of High Blood Pressure in Adults. *Circulation*. 2025. doi: 10.1161/circulationaha.125.075742

35.	Lloyd-Jones DM, Allen NB, Anderson CAM, Black T, Brewer LC, Foraker RE, Grandner MA, Lavretsky H, Perak AM, Sharma G, et al. Life’s Essential 8: Updating and Enhancing the American Heart Association’s Construct of Cardiovascular Health: A Presidential Advisory From the American Heart Association. *Circulation*. 2022;146:e18-e43. doi: 10.1161/CIR.0000000000001078
